# Supplementary material for: Modular one-pot assembly of CRISPR arrays enables library generation and reveals factors influencing crRNA biogenesis
Source: Nat Commun. 2019 Jul 3;10:2948. doi: 10.1038/s41467-019-10747-3 (PMC6610086; doi:10.1038/s41467-019-10747-3)
Supplement: Supplementary file 1 — Supplementary Information [file 41467_2019_10747_MOESM1_ESM.pdf]

Modular one-pot assembly of CRISPR arrays enables library generation and reveals factors influencing crRNA biogenesis

Liao et al.

SUPPLEMENTARY TABLES

Supplementary Table 1. Prior assembly methods to generate CRISPR arrays harboring multiple spacers. All methods rely on annealing two oligonucleotides to form individual subunits, and ligating the subunits individually or in combination into the base construct. Note that all prior methods used existing portions of the repeat and/or spacer as the assembly junctions, resulting in these sequences being coupled and

Ref.	Brief Description	Potential limitations
1,2	Type I arrays assembled through sequential insertion of repeat-spacer subunits. Insertion through the reuse of two restriction sites.	Repeat-spacer subunits must be added sequentially.
3	Type II arrays assembled through sequential insertion of spacer-repeat subunits. Insertion into a non-targeting spacer.	Spacer-repeat subunits must be added sequentially.
4–6	Type V-A, VI arrays assembled using repeat-spacer-repeat subunits. Junctions all involve 5’ overhangs and span the entire repeat and a few nts of the spacer.	Requires long and expensive oligonucleotides, depends on separate steps for array assembly and ligation, and would lead to extensive mis-pairing through the repeat portion of the overhangs. Incompatible with library generation because using part of spacer in the overhang.
7	Type V-A arrays assembled using repeat-spacer subunits. Junctions all involve 5’ overhangs and span different parts of the repeat.	Requires separate steps for array assembly and ligation, is restrained by the number of highly dissimilar overhangs that can be created from the repeat, and new junctions must be identified and tested when using repeats from different CRISPR-Cas systems.

Supplementary Table 2. Key resources used in this work.

Reagent or Resource	Source	Identifier
**Mammalian cell lines**		
HEK293T cells	Smyth lab (HIRI)	Supplementary Table 3
**Bacterial strains**		
E. coli BW25113 ΔCRISPR Δ*lacIZYA*	Supplementary Table 3	Supplementary Table 3
S. cerevisiae strain YPH500	Supplementary Table 3	Supplementary Table 3
E. coli TOP10	Supplementary Table 3	Supplementary Table 3
*E. col*i DH5α	Supplementary Table 3	Supplementary Table 3
E. coli Novablue	Supplementary Table 3	Supplementary Table 3
E. coli Tg1	Supplementary Table 3	Supplementary Table 3
**Chemicals, Peptides, and Recombinant Proteins**		
Q5 DNA Hot Start High-Fidelity DNA Polymerase	New England Biolabs	M0493
BsmBI	New England Biolabs	R0580S
BsaI-HF	New England Biolabs	R3535S
T4 DNA ligase	New England Biolab	M0202T
**Critical Commercial Assays**		
MyTXTL	Arbor Biosciences	507024
ZymoPURE Plasmid Midi Prep Kit	Zymo Research	D4200
NucleoSpin Plasmid EasyPure	Macherey-Nagel	740727.25
Gibson Assembly Cloning Kit	New England Biolabs	E5510S
NEBuilder HiFi DNA Assembly Cloning Kit	New England Biolabs	E5520S
Q5 Site-Directed Mutagenesis Kit	New England Biolabs	E0554S
Direct-zol RNA MiniPrep Plus w/ TRI Reagent	Zymo Research	R2071
TURBO DNA-free Kit	Invitrogen	AM1907
T4 Polynucleotide Kinase	New England Biolabs	M0201S
RNA Clean and Concentrator Kit	Zymo Research	R1015
Ribo-Zero rRNA Removal Kit (Bacteria)	Illumina	MRZMB126
NEBNext Multiplex Small RNA Library Prep Set for Illumina (Set 1)	New England Biolabs	E7300S
Select-a-Size DNA Clean & Concentrator (Spin-Column)	Zymo Research	D4080

**Deposited Data**		
RNA-seq analysis of processed FnCas12a arrays	Supplementary Table 3	Supplementary Table 3
**Oligonucleotides**		
Primers and cloning oligonucleotides	Supplementary Table 4	Supplementary Table 4
**Recombinant DNA**		
Plasmids	Supplementary Table 3	Supplementary Table 3
**Software and Algorithms**		
Geneious 10.2.3	Geneious	N/A
**Other**		
Detailed protocol for array assembly	Supplementary Methods	Supplementary Methods

Supplementary Table 3. List of microbial strains, plasmids, and NGS data used in this work.

Strain	Genotype	Source
E. coli DH5α	F– Φ80*lac*ZΔM15 Δ(*lac*ZYA-*arg*F) U169 *rec*A1 *end*A1 *hsd*R17 (rK–, mK+) *pho*A *sup*E44 λ– *thi*-1 *gyr*A96 *rel*A1	New England Biolabs
E. coli Novablue	K-12 *endA1 hsdR17* (rK12– mK12+) *supE44 thi-1 recA1 gyrA96 relA1 lac F'*[*proA+B+ lacIqZΔM15::*Tn*10*] (Tet*R*)	Merck KGaA
E. coli Top 10	F– *mcr*A Δ(*mrr-hsd*RMS-*mcr*BC) Φ80*lac*ZΔM15 Δ*lac*X74 *rec*A1 *ara*D139 Δ(*ara leu*) 7697 *gal*U *gal*K *rps*L (StrR) *end*A1 *nup*G	Invitrogen
E. coli BW25113 ΔCRISPRΔ*cat*Δ*lacIZYA*Δ*cat*	BW25113 ΔCRISPR-Cas Δ*lacIZYA*	Ref. 8
S. cerevisiae strain YPH500	a, ura3-52, lys2-801, ade2-101, trp1D63, his3D200, leu2D1	Ref. 9
E.coli Tg1	K-12 *supE thi-1* Δ*(lac-proAB)* Δ*(mcrB-hsdSM)5, (rK-mK-)*	Lucigen

Plasmid name	Plasmid sequence	CB ID	Internal ID	Source
pFnCpf1GG	https://benchling.com/s/seq-O1opLF5MXwGcHQtzllWO	pCB858	pCL146	This study
pcF-1	https://benchling.com/s/seq-BqQsWGDvM7sVahU4qcO5	pCB859	pCL244	This study
pcF-2	https://benchling.com/s/seq-FDKFhs4mFALXnGeDy8EU	pCB860	pCL246	This study
pcF-3	https://benchling.com/s/seq-fhYC7AsMWYfdfx1HMZfU	pCB861	pCL247	This study
pcF-1/2/3	https://benchling.com/s/seq-9Z8RFXOnoj3k0mqzDdnu	pCB862	pCL287	This study
pcF-1/3/2	https://benchling.com/s/seq-rGUW24L4UHDS6e34vipY	pCB863	pCL235	This study
pcF-2/1/3	https://benchling.com/s/seq-uE5l1UpP9pZJyePfst3d	pCB864	pCL285	This study
pcF-3/1/2	https://benchling.com/s/seq-8usT9w3z55tRztTEGOxT	pCB865	pCL289	This study
pcF-2/3/1	https://benchling.com/s/seq-ZSrBUTJtNqtFwCV5uatj	pCB866	pCL231	This study
pcF-3/2/1	https://benchling.com/s/seq-1MHJwpgRZQ6t6ikenwHh	pCB867	pCL290	This study
ptF-1	https://benchling.com/s/seq-xtfNRHv4vaOubss3sIWt	pCB868	pCL217	This study
ptF-2	https://benchling.com/s/seq-PfrOtTYNycHasm9iIZ68	pCB869	pCL213	This study
ptF-3	https://benchling.com/s/seq-cKaZqrd2y2Wxmr594X34	pCB870	pCL215	This study
pFncpf1_7_spacer_array	https://benchling.com/s/seq-DVmfEAwSCwMPCQUPmarr	pCB871	pCL226	This study
pBAD33_PJ23108_FnCas12a	https://benchling.com/s/seq-ITIiR0BNUXFHkHc8ZKBT	pCB872	pCL164	This study
pBAD33_PJ23108_1'2'3'dFnCas12a	https://benchling.com/s/seq-QL6lm0gCsSZor0yg9Ssi	pCB873	pCL131	This study

pBAD33_PJ23108_1'2'dFnCas12a	https://benchling.com/s/seq-GhbFITdNea265yFyI9JU	pCB874	pRL205	Ref. 8
pBAD33_PJ23108_1'dFnCas12a	https://benchling.com/s/seq-JdKfoI0GqLxSsY9Tbu8U	pCB875	pRL256	This study
pBAD33_PJ23108_2'dFnCas12a	https://benchling.com/s/seq-kZDPQNsSI7kNVAnXwOb3	pCB876	pRL257	This study
pBAD33_PJ23108_3'dFnCas12a	https://benchling.com/s/seq-EwHQDfeMxLmgcvf4VJJz	pCB877	pRS29	This study
pcF-4	https://benchling.com/s/seq-RnO4zTZbJfp3fPi4c9Ri	pCB878	pCL244	This study
pcF-5	https://benchling.com/s/seq-TPxkS31EqBFsQkeUhIPt	pCB879	pCL253	This study
pcF-6	https://benchling.com/s/seq-pMFLjBOhTC6kUnrYIBTS	pCB880	pRS85	This study
pcF-4/5/6	https://benchling.com/s/seq-eIemyR9TfE8XvK18erVM	pCB881	RS97	This study
pcF-4/6/5	https://benchling.com/s/seq-a9PaQlqgje7E1pT7XQD8	pCB882	RS98	This study
pcF-5/4/6	https://benchling.com/s/seq-dfGp2Fg1ci9TYOsTpu8a	pCB883	RS99	This study
pt-r1	https://benchling.com/s/seq-46hGdXyv5FrKlM2MB3Nm	pCB884	pRL131	Ref. 8
pt-r2	https://benchling.com/s/seq-5NIcgU3yMneTBAanm3ma	pCB885	pCL227	This study
pt-r3	https://benchling.com/s/seq-iGBF9P2G9M9SDLGinxMT	pCB208	pCB208	This study
pcFar	https://benchling.com/s/seq-XT0al2JDjBqMSqhLoSOh	pCB886	pFT25	This study
pCC22FnGGextr	https://benchling.com/s/seq-34AXxhR6fcI33U3R6Bkv	pCB887	pFT31	This study
PcFa-nt	https://benchling.com/s/seq-EbtRAqMHmUjIveuxlxjw	pCB888	pFT38	This study
PcFa-1	https://benchling.com/s/seq-zhZIvMNaWkmmeV42QsSa	pCB889	pFT32	This study
pcFa-2	https://benchling.com/s/seq-Q3o9ZGG47LDrh1ySSzlV	pCB890	pFT33	This study
pcFa-3	https://benchling.com/s/seq-OytHrrWEvq19eSPNXojp	pCB891	pFT35	This study
PcFa-1/2/3	https://benchling.com/s/seq-2q1w5eqeFV3Vy3I6IzoA	pCB892	pFT47	This study
PcFa-2/3/1	https://benchling.com/s/seq-o9QYqkw4atSd97po6W9E	pCB893	pFT48	This study
pcFa-3/1/2	https://benchling.com/s/seq-ieHG3E6Uy5rIoExQ46Mf	pCB894	pFT49	This study
pRS414	https://benchling.com/s/seq-IzuEoZARsUd8lmve9Rcp	pCB895	pRS414	Ref. 9
pCas9GG	https://benchling.com/s/seq-0JEUqzugSXYAWSzm6geL	pCB896	pCL239	This study
pCas9	https://benchling.com/s/seq-0NHTZbrxxDMvR9x785fi	pCB339	pCL284	Addgene #42876
pcS-1	https://benchling.com/s/seq-1EMEzsdQ3IzOySmQnkBq	pCB897	pFT26	This study
pcS-2	https://benchling.com/s/seq-EpCjwu5GO5dvG79uoMvg	pCB898	pFT27	This study
pcS-3	https://benchling.com/s/seq-TadGSEv51you4OEbL2b6	pCB899	pFT28	This study

pcS-1/2/3	https://benchling.com/s/seq-oysEYdpn8pZWZwdNTboJ	pCB900	pFT29	This study
pCas9GG_nt	https://benchling.com/s/seq-gdFq7GIPgYt6zgwAkUOy	pCB901	pFT30	This study
ptS-1	https://benchling.com/s/seq-46hGdXyv5FrKlM2MB3Nm	pCB902	pCL103 (pRL131)	Ref. 8
ptS-2	https://benchling.com/s/seq-9jAoErvusZrJX8cNfNiF	pCB903	pCL181	This study
ptS-3	https://benchling.com/s/seq-VcDmUpRRcZ62vIRDTQkZ	pCB904	pCL183	This study
pLsCas13aGG	https://benchling.com/s/seq-dfNbbatUBlz71XodynlG	pCB905	pFT50	This study
pBAD33_PJ23108_LsCas13a	https://benchling.com/s/seq-ccVpp4fLi8BDDbwuv7TJ	pCB906	pCL318	This study
p70a-deGFP	https://benchling.com/s/seq-tYmUX41asMH0heSSVGaJ	pCB907	pCL317 (pCSM254)	Ref. 10
ptL-nt	https://benchling.com/s/seq-y6t6fgj3nwWhc8h6TAa8	pCB908	pCL217	This study
ptL-1	https://benchling.com/s/seq-xXCmXtah3rTBBFAlaipD	pCB909	pFT62	This study
ptL-2	https://benchling.com/s/seq-DUADJ56gazIIwZEtUYY8	pCB910	pFT63	This study
ptL-3	https://benchling.com/s/seq-GoFs8F3tCd9w1PVEhXFd	pCB911	pFT64	This study
pcL-nt	https://benchling.com/s/seq-nvi4bKb8TiopXLf6Riks	pCB912	pFT57	This study
pcL-1	https://benchling.com/s/seq-kDiKu7V10wZ2C37stlvX	pCB913	pFT52	This study
pcL-2	https://benchling.com/s/seq-CRAsHBMxehZfo1nRgTs3	pCB914	pFT53	This study
pcL-3	https://benchling.com/s/seq-BgflBmOcM8cyPQ11hmqh	pCB915	pFT55	This study
pcL-3/2/1	https://benchling.com/s/seq-522svi85LWMxx2nbQRSM	pCB918	pCL323	This study
pta	https://benchling.com/s/seq-46hGdXyv5FrKlM2MB3Nm	pCB919	pCL103 (pRL131)	Ref. 8
ptb	https://benchling.com/s/seq-5NIcgU3yMneTBAanm3ma	pCB920	pCL227	This study
pCas9FnCpf1GG	https://benchling.com/s/seq-3E0VYBMEgCVGmnSg663k	pCB921	pCL241	This study
pc7	https://benchling.com/s/seq-bkXSEGQKTRx0wcXYlrJW	pCB922	pCL251	This study
pc8	https://benchling.com/s/seq-dTldefq3pnJBeWS1rP69	pCB923	pCL301	This study
pc9	https://benchling.com/s/seq-cpaksqKZZETJHUPKePlN	pCB924	pCL253	This study
pc10	https://benchling.com/s/seq-ZW9gxzpuNWNmQfVaerBe	pCB925	pCL255	This study
pc7/8/9/10	https://benchling.com/s/seq-1MLCffsHqs9eAY68MP6l	pCB926	pCL303	This study
pc8/7/9/10	https://benchling.com/s/seq-sTnkf5XX5YowwvlTaq7N	pCB927	pFT59	This study
pc-nt	https://benchling.com/s/seq-u6Kzd7OCuLz1iqwe7LvW	pCB928	pCL243	This study
p-on	https://benchling.com/s/seq-ydDcm02VuDDCzMELRNvf	pCB929	pCL331	This study

p-off1	https://benchling.com/s/seq-2oimoOvw66wdxSEuMLFI	pCB930	pCL333	This study
p-off2	https://benchling.com/s/seq-XKicEoHQz7CkoBXiM3Cf	pCB931	pCL334	This study
poffGG	https://benchling.com/s/seq-XhDTZsDfObs2Ab1Mn3A8	pCB932	pFT68	This study
psg	https://benchling.com/s/seq-iOk8Wh1COy0PTisEDNad	pCB933	pFT70	This study
pb1	https://benchling.com/s/seq-1gDCZDRC0fq9zqSICRIR	pCB934	pRS136	This study
pb2	https://benchling.com/s/seq-tjy46a6HcOvaaFSfepCB	pCB935	pRS137	This study
psg/b1/b2	https://benchling.com/s/seq-qwLH298BVtaCKtzkRXIG	pCB936	pCL339	This study
psg/b2/b1	https://benchling.com/s/seq-4U20HFzAIfZJBB7D2Et6	pCB937	pCL341	This study
pCas9-notracr	https://benchling.com/s/seq-VjwM3Ur4nCuOJM6NoJzS	pCB938	pSPC026	This study
pcF-1	https://benchling.com/s/seq-BqQsWGDvM7sVahU4qcO5	pCB939	pCL244	This study
pcF-1'	https://benchling.com/s/seq-R09cVII06wrU8ET8zTr9	pCB940	pSD17	This study
pcF-2	https://benchling.com/s/seq-FDKFhs4mFALXnGeDy8EU	pCB941	pCL246	This study
pcF-2'	https://benchling.com/s/seq-qu5MGMzTH2RhPnNELomu	pCB942	pFT45	This study
pcF-3	https://benchling.com/s/seq-fhYC7AsMWYfdfx1HMZfU	pCB943	pCL247	This study
pcF-3'	https://benchling.com/s/seq-RFP6ms4pWrRuUFNbTLed	pCB944	pFT46	This study
ptA-1 same as ptF1	https://benchling.com/s/seq-xtfNRHv4vaOubss3sIWt	pCB945	pCL217	This study
ptA-2 same as ptF2	https://benchling.com/s/seq-PfrOtTYNycHasm9iIZ68	pCB946	pCL213	This study
ptA-3 same as ptF3	https://benchling.com/s/seq-cKaZqrd2y2Wxmr594X34	pCB947	pCL215	This study
pcF-1/3/2r	https://benchling.com/s/seq-yJyqN0p1GujdmWHRwcdk	pCB948	pFT74	This study
pcF-2/3/1r	https://benchling.com/s/seq-gABsCjcTzZO0DYvCxBBi	pCB949	pFT69	This study
pAsCpf1GG	https://benchling.com/s/seq-DtHPCWNS7oROcxFNxXmS	pCB950	pCL247	This study
pBAD33_PJ23108_AsCas12a	https://benchling.com/s/seq-NIW6idz1n8rgEIXOgAIU	pCB951	pRS72	This study
pcA-1	https://benchling.com/s/seq-ybFkXVaat2y9rV0q6NkR	pCB952	pRS51	This study
pcA-2	https://benchling.com/s/seq-gqFW9DkQD5c0MfE52zrW	pCB953	pRS52	This study
pcA-3	https://benchling.com/s/seq-GCDLYFMvETLmCSg8arhT	pCB954	pRS53	This study
pcA-1/2/3	https://benchling.com/s/seq-eALq3dqVtCYNR3WbyV9H	pCB955	pRS63	This study
pcA-nt	https://benchling.com/s/seq-pZbNB9gC2YMuDYS6EIv9	pCB956	pRS49	This study
pDEST-hisMBP-AsCpf1-EC	https://benchling.com/s/seq-nexV8vWDTenrjfLuPX2E	pCB957	pCL212	Addgene #79007

pC003_LshC2c2	https://benchling.com/s/seq-BOkN0XzeSQ1F6Vt10ClH	pCB958	pCL298	Addgene #79152
ptF-1m1	https://benchling.com/s/seq-kTeh5y2PePN2I9ZuE8MM	CBS-232	CL429	This study
ptF-1m2	https://benchling.com/s/seq-PBx4222jQhdw15maxu9l	CBS-233	CL430	This study
ptF-1m5	https://benchling.com/s/seq-N2uraFcK3UEZBFeul00r	CBS-234	CL431	This study
ptF-1m4	https://benchling.com/s/seq-4PyOzRhc0pTceYfQqQii	CBS-235	CL432	This study
ptF-1m3	https://benchling.com/s/seq-CZJua6OiTh0kU63exj0B	CBS-236	CL433	This study
ptF-1m5'	https://benchling.com/s/seq-wgB97ugGJVLVEhBztBlj	CBS-237	CL434	This study
ptF-1m4'	https://benchling.com/s/seq-gj0QtB0nlMRlOqS4XoaI	CBS-238	CL435	This study
pcF-2/3/1m1	https://benchling.com/s/seq-VECNkCINWmfkadyBenxX	CBS-239	CL436	This study
pcF-2/3/1m2	https://benchling.com/s/seq-keZUX9tCur69z6LXuibt	CBS-240	CL437	This study
pcF-2/3/1m5	https://benchling.com/s/seq-JvXX6kKG1U6c8MpPjbst	CBS-241	CL438	This study
pcF-2/3/1m4	https://benchling.com/s/seq-Ujeijppa6iGssO9aHQpo	CBS-242	CL439	This study
pcF-2/3/1m3	https://benchling.com/s/seq-w34cDmgFf3x8SBvEYANJ	CBS-243	CL440	This study
pcF-2/3/1m5'	https://benchling.com/s/seq-gj3KYYCxjH3VRlVLwl1e	CBS-244	CL441	This study
pcF-2/3/1m4'	https://benchling.com/s/seq-4C0UqIp8tM9WjqYmCCPr	CBS-245	CL442	This study
pcF-1/3/2NR	https://benchling.com/s/seq-gRqhcIprYVzdbPunCggf	CBS-456	CL455	This study
pMZ-7spacerarrayWTterminalrepeat	https://benchling.com/s/seq-ubxXHzQoNgZCuaKGEwvN	CBS-457	CL453	This study
pMZ-7spacerarrayNativeterminalrepeat	https://benchling.com/s/seq-2E4NSKaFpT1pFZr2aZAc	CBS-458	CL454	This study
pUA66-targforunintent	https://benchling.com/s/seq-8Sbbe2tuVs6H2vWkZo1W	CBS-459	CL456	This study

Samples subjected for NGS	Description	Source	BioSample accessions	NCBI data deposition link
CL1	crRNA from array Fncpf1_7_spacer_array from TXTL	This study	SAMN09049816	https://www.ncbi.nlm.nih.gov/sra/SRP144980
CL2	crRNA from 3-spacer array cF-2/3/1	This study	SAMN09049817	https://www.ncbi.nlm.nih.gov/sra/SRP144980
CL3	crRNA from 3-spacer array cF-1/3/2	This study	SAMN09049818	https://www.ncbi.nlm.nih.gov/sra/SRP144980
Sample_2_R1	library of CRISPR-array	This study	SAMN10236521	https://www.ncbi.nlm.nih.gov/sra/PRJNA496034
Sample_2_R2	library of CRISPR-array	This study	SAMN10236522	https://www.ncbi.nlm.nih.gov/sra/PRJNA496034
conserved	crRNA from array Fncpf1_7_spacer_array with consensus repeat as terminal repeat from mammalian cells	This study	SAMN11027815	https://www.ncbi.nlm.nih.gov/sra/PRJNA454865
native	crRNA from array Fncpf1_7_spacer_array with native terminal repeat from mammalian cells	This study	SAMN11027816	https://www.ncbi.nlm.nih.gov/sra/PRJNA454865

Supplementary Table 4. Sequences of CRISPR arrays, oligonucleotides used in this work, and a list of junctions to make large arrays. The specific junction sequences are noted for each multi-spacer CRISPR array. Names correspond to those in Supplementary Table 3.

Array	Sequence of array
Fncpf1_7_spacer_array	GTCTAAGAACTTTAAATAATTTCTACTGTTGTAGATGAAATCGAAGGTGAAGGTGAAGGTCGTTCTGTCTAAGAACTTTAAATAATTTCTACTGTTGTAGATGAAGACGGTGGTGTTGTTACCGTTACTGGCGTCTAAGAACTTTAAATAATTTCTACTGTTGTAGATTTTACGATGCCATTGGGATATATCAAAGAAGTCTAAGAACTTTAAATAATTTCTACTGTTGTAGATGGCTGGCGCGAGCCCCTGATGCTCTTTACAGTCTAAGAACTTTAAATAATTTCTACTGTTGTAGATTTGACAGCTAGCTCAGTCCTAGGTATGCTGGTCTAAGAACTTTAAATAATTTCTACTGTTGTAGATACCTCGAGGGGATCCTCTAGATTTAAGAGTGTCTAAGAACTTTAAATAATTTCTACTGTTGTAGATACATACGAGCCGGAAGCATAAAGTGTAACGGTCTAAGAACTTTAAATAATTTCTACTGTTGTAGAT
cF-1	GTCTAAGAACTTTAAATAATTTCTACTGTTGTAGATCTTTACACTTTATGCTTCCGGCTCGATCGAGTCTAAGAACTTTAAATAATTTCTACTGTTGTAGAT
cF-2	GTCTAAGAACTTTAAATAATTTCTACTGTTGTAGATCGTATGTTGCATCACCTTCACCCTCTAACGGTCTAAGAACTTTAAATAATTTCTACTGTTGTAGAT
cF-3	GTCTAAGAACTTTAAATAATTTCTACTGTTGTAGATCTGTACATAACCTTCGGGCATGGCACAACGGTCTAAGAACTTTAAATAATTTCTACTGTTGTAGAT
cF-1/2/3	GTCTAAGAACTTTAAATAATTTCTACTGTTGTAGATCTTTACACTTTATGCTTCCGGCTCGTGCTGGTCTAAGAACTTTAAATAATTTCTACTGTTGTAGATCGTATGTTGCATCACCTTCACCCTCTGAGTGTCTAAGAACTTTAAATAATTTCTACTGTTGTAGATCTGTACATAACCTTCGGGCATGGCACAACGGTCTAAGAACTTTAAATAATTTCTACTGTTGTAGAT
cF-1/3/2	GTCTAAGAACTTTAAATAATTTCTACTGTTGTAGATCTTTACACTTTATGCTTCCGGCTCGTGCTGGTCTAAGAACTTTAAATAATTTCTACTGTTGTAGATCTGTACATAACCTTCGGGCATGGCACGAGTGTCTAAGAACTTTAAATAATTTCTACTGTTGTAGATCGTATGTTGCATCACCTTCACCCTCTAACGGTCTAAGAACTTTAAATAATTTCTACTGTTGTAGAT
cF-2/1/3	GTCTAAGAACTTTAAATAATTTCTACTGTTGTAGATCGTATGTTGCATCACCTTCACCCTCTGCTGGTCTAAGAACTTTAAATAATTTCTACTGTTGTAGATCTTTACACTTTATGCTTCCGGCTCGTGAGTGTCTAAGAACTTTAAATAATTTCTACTGTTGTAGATCTGTACATAACCTTCGGGCATGGCACAACGGTCTAAGAACTTTAAATAATTTCTACTGTTGTAGAT
cF-3/1/2	GTCTAAGAACTTTAAATAATTTCTACTGTTGTAGATCTGTACATAACCTTCGGGCATGGCACGCTGGTCTAAGAACTTTAAATAATTTCTACTGTTGTAGATCTTTACACTTTATGCTTCCGGCTCGTGAGTGTCTAAGAACTTTAAATAATTTCTACTGTTGTAGATCGTATGTTGCATCACCTTCACCCTCTAACGGTCTAAGAACTTTAAATAATTTCTACTGTTGTAGAT
cF-2/3/1	GTCTAAGAACTTTAAATAATTTCTACTGTTGTAGATCGTATGTTGCATCACCTTCACCCTCTGCTGGTCTAAGAACTTTAAATAATTTCTACTGTTGTAGATCTGTACATAACCTTCGGGCATGGCACGAGTGTCTAAGAACTTTAAATAATTTCTACTGTTGTAGATCTTTACACTTTATGCTTCCGGCTCGTAACGGTCTAAGAACTTTAAATAATTTCTACTGTTGTAGAT
cF-3/2/1	GTCTAAGAACTTTAAATAATTTCTACTGTTGTAGATCTGTACATAACCTTCGGGCATGGCACGCTGGTCTAAGAACTTTAAATAATTTCTACTGTTGTAGATCGTATGTTGCATCACCTTCACCCTCTGAGTGTCTAAGAACTTTAAATAATTTCTACTGTTGTAGATCTTTACACTTTATGCTTCCGGCTCGTAACGGTCTAAGAACTTTAAATAATTTCTACTGTTGTAGAT
cF-4	GTCTAAGAACTTTAAATAATTTCTACTGTTGTAGATCTTTACACTTTATGCTTCCGGCTCGATCGAGTCTAAGAACTTTAAATAATTTCTACTGTTGTAGAT
cF-5	GTCTAAGAACTTTAAATAATTTCTACTGTTGTAGATGTCGAGTGCAAAACCTTTCGCGGTATAACGGTCTAAGAACTTTAAATAATTTCTACTGTTGTAGAT

cF-6	GTCTAAGAACTTTAAATAATTTCTACTGTTGTAGATTCCATACCCGTTTTTTTGGATGGAGTAACGGTCTAAGAACTTTAAATAATTTCTACTGTTGTAGAT
cF-4/5/6	GTCTAAGAACTTTAAATAATTTCTACTGTTGTAGATCTTTACACTTTATGCTTCCGGCTCGTGCTGGTCTAAGAACTTTAAATAATTTCTACTGTTGTAGATGTCGAGTGCAAAACCTTTCGCGGTATGAGTGTCTAAGAACTTTAAATAATTTCTACTGTTGTAGATTCCATACCCGTTTTTTTGGATGGAGTAACGGTCTAAGAACTTTAAATAATTTCTACTGTTGTAGAT
cF-4/6/5	GTCTAAGAACTTTAAATAATTTCTACTGTTGTAGATCTTTACACTTTATGCTTCCGGCTCGTGCTGGTCTAAGAACTTTAAATAATTTCTACTGTTGTAGATTCCATACCCGTTTTTTTGGATGGAGTGAGTGTCTAAGAACTTTAAATAATTTCTACTGTTGTAGATGTCGAGTGCAAAACCTTTCGCGGTATAACGGTCTAAGAACTTTAAATAATTTCTACTGTTGTAGAT
cF-5/4/6	GTCTAAGAACTTTAAATAATTTCTACTGTTGTAGATGTCGAGTGCAAAACCTTTCGCGGTATGCTGGTCTAAGAACTTTAAATAATTTCTACTGTTGTAGATCTTTACACTTTATGCTTCCGGCTCGTGAGTGTCTAAGAACTTTAAATAATTTCTACTGTTGTAGATTCCATACCCGTTTTTTTGGATGGAGTAACGGTCTAAGAACTTTAAATAATTTCTACTGTTGTAGAT
cFa-1	GTCTAAGAACTTTAAATAATTTCTACTGTTGTAGATGTGCTGACACATACAGGCATATATATAACGGTCTAAGAACTTTAAATAATTTCTACTGTTGTAGAT
cFa-2	GTCTAAGAACTTTAAATAATTTCTACTGTTGTAGATGAGTCCTCACTCTGAATTCGATATCAAACGGTCTAAGAACTTTAAATAATTTCTACTGTTGTAGAT
cFa-3	GTCTAAGAACTTTAAATAATTTCTACTGTTGTAGATCCAGTCACGACGTTGTAAAACGACGGAACGGTCTAAGAACTTTAAATAATTTCTACTGTTGTAGAT
cFa-1/2/3	GTCTAAGAACTTTAAATAATTTCTACTGTTGTAGATGTGCTGACACATACAGGCATATATATGCTGGTCTAAGAACTTTAAATAATTTCTACTGTTGTAGATGAGTCCTCACTCTGAATTCGATATCAGAGTGTCTAAGAACTTTAAATAATTTCTACTGTTGTAGATCCAGTCACGACGTTGTAAAACGACGGAACGGTCTAAGAACTTTAAATAATTTCTACTGTTGTAGAT
cFa-2/3/1	GTCTAAGAACTTTAAATAATTTCTACTGTTGTAGATGAGTCCTCACTCTGAATTCGATATCAGCTGGTCTAAGAACTTTAAATAATTTCTACTGTTGTAGATCCAGTCACGACGTTGTAAAACGACGGGAGTGTCTAAGAACTTTAAATAATTTCTACTGTTGTAGATGTGCTGACACATACAGGCATATATATAACGGTCTAAGAACTTTAAATAATTTCTACTGTTGTAGAT
cFa-3/1/2	GTCTAAGAACTTTAAATAATTTCTACTGTTGTAGATCCAGTCACGACGTTGTAAAACGACGGGCTGGTCTAAGAACTTTAAATAATTTCTACTGTTGTAGATGTGCTGACACATACAGGCATATATATGAGTGTCTAAGAACTTTAAATAATTTCTACTGTTGTAGATGAGTCCTCACTCTGAATTCGATATCAAACGGTCTAAGAACTTTAAATAATTTCTACTGTTGTAGAT
cS-1	GTTTTAGAGCTATGCTGTTTTGAATGGTCCCAAAACCGCATTTATGCTTCCGGCTCGTATGTTGTGGTTTTAGAGCTATGCTGTTTTGAATGGTCCCAAAAC
cS-2	GTTTTAGAGCTATGCTGTTTTGAATGGTCCCAAAACCGCACAAAGGTGGTCCGCTGCCGTTCGCTTGTTTTAGAGCTATGCTGTTTTGAATGGTCCCAAAAC
cS-3	GTTTTAGAGCTATGCTGTTTTGAATGGTCCCAAAACCGCAATGGCTAAAAAACCGGTTCAGCTGCCGTTTTAGAGCTATGCTGTTTTGAATGGTCCCAAAAC
cS-1/2/3	GTTTTAGAGCTATGCTGTTTTGAATGGTCCCAAAACCGCATTTATGCTTCCGGCTCGTATGTTGTGGTTTTAGAGCTATGCTGTTTTGAATGGTCCCAAAACCCCTCAAAGGTGGTCCGCTGCCGTTCGCTTGTTTTAGAGCTATGCTGTTTTGAATGGTCCCAAAACGAGTATGGCTAAAAAACCGGTTCAGCTGCCGTTTTAGAGCTATGCTGTTTTGAATGGTCCCAAAAC
cL-1	GATATAGACCACCCCAATATCGAAGGGGACTAAAACTAGTCCGGGATGTCAGCCGGGTGTTTAAACGGATATAGACCACCCCAATATCGAAGGGGACTAAAAC
cL-2	GATATAGACCACCCCAATATCGAAGGGGACTAAAACGTAGATGAACTCACCGTCTTGCAGGGAAACGGATATAGACCACCCCAATATCGAAGGGGACTAAAAC
cL-3	GATATAGACCACCCCAATATCGAAGGGGACTAAAACAGGCGATTAAGTTGGGTAACGCCAGGGAACGGATATAGACCACCCCAATATCGAAGGGGACTAAAAC

cL-3/2/1	GATATAGACCACCCCAATATCGAAGGGGACTAAAACAGGCGATTAAGTTGGGTAACGCCAGGGGCTGGATATAGACCACCCCAATATCGAAGGGGACTAAAACGTAGATGAACTCACCGTCTTGCAGGGAGAGTGATATAGACCACCCCAATATCGAAGGGGACTAAAACTAGTCCGGGATGTCAGCCGGGTGTTTAAACGGATATAGACCACCCCAATATCGAAGGGGACTAAAAC
c7	GTTTTAGAGCTATGCTGTTTTGAATGGTCCCAAAACCCCTTTTATGCTTCCGGCTCGTATGTTGTGGTTTTAGAGCTATGCTGTTTTGAATGGTCCCAAAACAACGGTCTAAGAACTTTAAATAATTTCTACTGTTGTAGAT
c8	GTTTTAGAGCTATGCTGTTTTGAATGGTCCCAAAACCCCTTTTATGCTTCCGGCTCGTATGTTGTGGTTTTAGAGCTATGCTGTTTTGAATGGTCCCAAAACAACGGTCTAAGAACTTTAAATAATTTCTACTGTTGTAGAT
c9	GTTTTAGAGCTATGCTGTTTTGAATGGTCCCAAAACCCCTGTCTAAGAACTTTAAATAATTTCTACTGTTGTAGATCTTTACACTTTATGCTTCCGGCTCGTAACGGTCTAAGAACTTTAAATAATTTCTACTGTTGTAGAT
c10	GTTTTAGAGCTATGCTGTTTTGAATGGTCCCAAAACCCCTGTCTAAGAACTTTAAATAATTTCTACTGTTGTAGATGTCGAGTGCAAAACCTTTCGCGGTATAACGGTCTAAGAACTTTAAATAATTTCTACTGTTGTAGAT
c7/8/9/10	GTTTTAGAGCTATGCTGTTTTGAATGGTCCCAAAACCCCTTTTATGCTTCCGGCTCGTATGTTGTGGTTTTAGAGCTATGCTGTTTTGAATGGTCCCAAAACTACACTTTCGCGGTATGGCATGATAGCGCCGTTTTAGAGCTATGCTGTTTTGAATGGTCCCAAAACGCTGGTCTAAGAACTTTAAATAATTTCTACTGTTGTAGATCTTTACACTTTATGCTTCCGGCTCGTGAGTGTCTAAGAACTTTAAATAATTTCTACTGTTGTAGATGTCGAGTGCAAAACCTTTCGCGGTATAACGGTCTAAGAACTTTAAATAATTTCTACTGTTGTAGAT
c8/7/9/10	GTTTTAGAGCTATGCTGTTTTGAATGGTCCCAAAACCCCTCTTTCGCGGTATGGCATGATAGCGCCGTTTTAGAGCTATGCTGTTTTGAATGGTCCCAAAACTACATTTATGCTTCCGGCTCGTATGTTGTGGTTTTAGAGCTATGCTGTTTTGAATGGTCCCAAAACGCTGGTCTAAGAACTTTAAATAATTTCTACTGTTGTAGATCTTTACACTTTATGCTTCCGGCTCGTGAGTGTCTAAGAACTTTAAATAATTTCTACTGTTGTAGATGTCGAGTGCAAAACCTTTCGCGGTATAACGGTCTAAGAACTTTAAATAATTTCTACTGTTGTAGAT
sg	TGGATGGAGGAATGAGGAGTGTTTTAGAGCTAGAAATAGCAAGTTAAAATAAGGCTAGTCCGAACGGTCTAAGAACTTTAAATAATTTCTACTGTTGTAGAT
b1	GTCTAAGAACTTTAAATAATTTCTACTGTTGTAGATCCACTCCTCATCCCTCCATCCCCTCAAACGGTCTAAGAACTTTAAATAATTTCTACTGTTGTAGAT
b2	GTCTAAGAACTTTAAATAATTTCTACTGTTGTAGATTGTACCCACTCCTCATTCCTCCCTCCAACGGTCTAAGAACTTTAAATAATTTCTACTGTTGTAGAT
sg/b1/b2	TGGATGGAGGAATGAGGAGTGTTTTAGAGCTAGAAATAGCAAGTTAAAATAAGGCTAGTCCGGAGTGTCTAAGAACTTTAAATAATTTCTACTGTTGTAGATCCACTCCTCATCCCTCCATCCCCTCATTCTGTCTAAGAACTTTAAATAATTTCTACTGTTGTAGATTGTACCCACTCCTCATTCCTCCCTCCAACGGTCTAAGAACTTTAAATAATTTCTACTGTTGTAGAT
sg/b2/b1	TGGATGGAGGAATGAGGAGTGTTTTAGAGCTAGAAATAGCAAGTTAAAATAAGGCTAGTCCGGAGTGTCTAAGAACTTTAAATAATTTCTACTGTTGTAGATTGTACCCACTCCTCATTCCTCCCTCCTTCTGTCTAAGAACTTTAAATAATTTCTACTGTTGTAGATCCACTCCTCATCCCTCCATCCCCTCAAACGGTCTAAGAACTTTAAATAATTTCTACTGTTGTAGAT
cA-t1	GTCAAAAGACCTTTTTAATTTCTACTCTTGTAGATCACTTTATGCTTCCGGCTCGTATGTTAACGGTCAAAAGACCTTTTTAATTTCTACTCTTGTAGAT
cA-t2	GTCAAAAGACCTTTTTAATTTCTACTCTTGTAGATTGTCAGTGGAGAGGGTGAAGGTGATGAACGGTCAAAAGACCTTTTTAATTTCTACTCTTGTAGAT
cA-t3	GTCAAAAGACCTTTTTAATTTCTACTCTTGTAGATAAGAGTGCCATGCCCGAAGGTTATGTAACGGTCAAAAGACCTTTTTAATTTCTACTCTTGTAGAT
cA-1/2/3	GTCAAAAGACCTTTTTAATTTCTACTCTTGTAGATCACTTTATGCTTCCGGCTCGTATGTTGCTGGTCAAAAGACCTTTTTAATTTCTACTCTTGTAGATTGTCAGTGGAGAGGGTGAAGGTGATGGAGTGTCAAAAGACCTTTTTAATTTCTACTCTTGTAGATAAGAGTGCCATGCCCGAAGGTTATGTAACGGTCAAAAGACCTTTTTAATTTCTACTCTTGTAGAT
cF-2/3/1m1	GTCTAAGAACTTTAAATAATTTCTACTGTTGTAGATCGTATGTTGCATCACCTTCACCCTCTGCTGGTCTAAGAACTTTAAATAATTTCTACTGTTGTAGATCTGTACATAACCTTCGGGCATGGCACGAGTGTCTAAGAACTTTAAATAATTTCTACTGTTGTAGATACATACACTTTATGCTTCCGGCTCGTAACGGTCTAAGAACTTTAAATAATTTCTACTGTTGTAGAT

cF-2/3/1m2	GTCTAAGAACTTTAAATAATTTCTACTGTTGTAGATCGTATGTTGCATCACCTTCACCCTCTGCTGGTCTAAGAACTTTAAATAATTTCTACTGTT<br>GTAGATCTGTACATAACCTTCGGGCATGGCACGAGTGTCTAAGAACTTTAAATAATTTCTACTGTTGTAGATCCTTACACTTTATGCTTCCGGCT<br>CGTAACGGTCTAAGAACTTTAAATAATTTCTACTGTTGTAGAT
cF-2/3/1m5	GTCTAAGAACTTTAAATAATTTCTACTGTTGTAGATCGTATGTTGCATCACCTTCACCCTCTGCTGGTCTAAGAACTTTAAATAATTTCTACTGTT<br>GTAGATCTGTACATAACCTTCGGGCATGGCACGAGTGTCTAAGAACTTTAAATAATTTCTACTGTTGTAGATCTTTACACTTTATGCTTCCGGCC<br>AGTAACGGTCTAAGAACTTTAAATAATTTCTACTGTTGTAGAT
cF-2/3/1m4	GTCTAAGAACTTTAAATAATTTCTACTGTTGTAGATCGTATGTTGCATCACCTTCACCCTCTGCTGGTCTAAGAACTTTAAATAATTTCTACTGTT<br>GTAGATCTGTACATAACCTTCGGGCATGGCACGAGTGTCTAAGAACTTTAAATAATTTCTACTGTTGTAGATCTTTACACTTTAGACTTCCGGCT<br>CGTAACGGTCTAAGAACTTTAAATAATTTCTACTGTTGTAGAT
cF-2/3/1m3	GTCTAAGAACTTTAAATAATTTCTACTGTTGTAGATCGTATGTTGCATCACCTTCACCCTCTGCTGGTCTAAGAACTTTAAATAATTTCTACTGTT<br>GTAGATCTGTACATAACCTTCGGGCATGGCACGAGTGTCTAAGAACTTTAAATAATTTCTACTGTTGTAGATCTTTAACCTTTATGCTTCCGGCT<br>CGTAACGGTCTAAGAACTTTAAATAATTTCTACTGTTGTAGAT
cF-2/3/1m5'	GTCTAAGAACTTTAAATAATTTCTACTGTTGTAGATCGTATGTTGCATCACCTTCACCCTCTGCTGGTCTAAGAACTTTAAATAATTTCTACTGTT<br>GTAGATCTGTACATAACCTTCGGGCATGGCACGAGTGTCTAAGAACTTTAAATAATTTCTACTGTTGTAGATCTTTACACTTTATGCTTCCGGCC<br>ATGAACGGTCTAAGAACTTTAAATAATTTCTACTGTTGTAGAT
cF-2/3/1m4'	GTCTAAGAACTTTAAATAATTTCTACTGTTGTAGATCGTATGTTGCATCACCTTCACCCTCTGCTGGTCTAAGAACTTTAAATAATTTCTACTGTT<br>GTAGATCTGTACATAACCTTCGGGCATGGCACGAGTGTCTAAGAACTTTAAATAATTTCTACTGTTGTAGATCTTTACACTCCAGACCCCCGGC<br>TCGTAACGGTCTAAGAACTTTAAATAATTTCTACTGTTGTAGAT
cF-1/3/2NR	GTCTAAGAACTTTAAATAATTTCTACTGTTGTAGATCTTTACACTTTATGCTTCCGGCTCGTGCTGGTCTAAGAACTTTAAATAATTTCTACTGTT<br>GTAGATCTGTACATAACCTTCGGGCATGGCACGAGTGTCTAAGAACTTTAAATAATTTCTACTGTTGTAGATCGTATGTTGCATCACCTTCACCC<br>TCTAACGGTCTAAGAACTTTAAATAATTTGTCTGTATATTATT

Oligo name (arrays)	Junction (orientation of the overhang) used
**Fncpf1_7_spacer_array**	**Junction (orientation): CCCT (5'), TTCT (3'), TGGC (5'), AGAA (3'), TACA (5'), GCTG (3'), GAGT (5'), AACG (3')**
NmRFPrepspa1GG_F	CCCTGTCTAAGAACTTTAAATAATTTCTACTGTTGTAGATGAAATCGAAGGTGAAGGTGAAGGTCGTTCT
NmRFPrepspa1GG_R	CGACCTTCACCTTCACCTTCGATTTCATCTACAACAGTAGAAATTATTTAAAGTTCTTAGAC
NmRFPrepspa2GG_F	GTCTAAGAACTTTAAATAATTTCTACTGTTGTAGATGAAGACGGTGGTGTTGTTACCGTTAC
NmRFPrepspa2GG_R	GCCAGTAACGGTAACAACACCACCGTCTTCATCTACAACAGTAGAAATTATTTAAAGTTCTTAGACAGAA
Nf7CmRrepspaGG_F	TGGCGTCTAAGAACTTTAAATAATTTCTACTGTTGTAGATTTTACGATGCCATTGGGATATATCAAAGAA
Nf7CmRrepspaGG_R	TTGATATATCCCAATGGCATCGTAAAATCTACAACAGTAGAAATTATTTAAAGTTCTTAGAC
KanRrepspaGGf5_F	GTCTAAGAACTTTAAATAATTTCTACTGTTGTAGATGGCTGGCGCGAGCCCCTGATGCTCTT
KanRrepspaGGf5_R	TGTAAAGAGCATCAGGGGCTCGCGCCAGCCATCTACAACAGTAGAAATTATTTAAAGTTCTTAGACTTCT
PJ231repspaGGf5_F	TACAGTCTAAGAACTTTAAATAATTTCTACTGTTGTAGATTTGACAGCTAGCTCAGTCCTAGGTATGCTG
PJ231repspaGGf5_R	ATACCTAGGACTGAGCTAGCTGTCAAATCTACAACAGTAGAAATTATTTAAAGTTCTTAGAC
repeatspacer2_F	GTCTAAGAACTTTAAATAATTTCTACTGTTGTAGATACCTCGAGGGGATCCTCTAGATTTAA

repeatspacer2_R	ACTCTTAAATCTAGAGGATCCCCTCGAGGTATCTACAACAGTAGAAATTATTTAAAGTTCTTAGACCAGC
repeatspacer3_F	GAGTGTCTAAGAACTTTAAATAATTTCTACTGTTGTAGATACATACGAGCCGGAAGCATAAAGTGT
repeatspacer3_R	CGTTACACTTTATGCTTCCGGCTCGTATGTATCTACAACAGTAGAAATTATTTAAAGTTCTTAGAC
**cF-1/2/3**	**CCCT (5'), GCTG (3'), GAGT (5'), AACG (3')**
Fnarrayspa1'_F	CCCTGTCTAAGAACTTTAAATAATTTCTACTGTTGTAGAT CTTTACACTTTATGCTTCCGGCTCGTGCTG
Fnarrayspa1'_R	ACGAGCCGGAAGCATAAAGTGTAAAG ATCTACAACAGTAGAAATTATTTAAAGTTCTTAGAC
Fnarrayspa52_F	GTCTAAGAACTTTAAATAATTTCTACTGTTGTAGATCGTATGTTGCATCACCTTCACCCTCT
Fnarrayspa52_R	ACTCAGAGGGTGAAGGTGATGCAACATACGATCTACAACAGTAGAAATTATTTAAAGTTCTTAGACCAGC
Fnarrayspa43_F	GAGTGTCTAAGAACTTTAAATAATTTCTACTGTTGTAGATCTGTACATAACCTTCGGGCATGGCAC
Fnarrayspa43_R	CGTTGTGCCATGCCCGAAGGTTATGTACAG ATCTACAACAGTAGAAATTATTTAAAGTTCTTAGAC
**cF-1/3/2**	**CCCT (5'), GCTG (3'), GAGT (5'), AACG (3')**
Fnarrayspa1'_F	CCCTGTCTAAGAACTTTAAATAATTTCTACTGTTGTAGATCTTTACACTTTATGCTTCCGGCTCGTGCTG
Fnarrayspa1'_R	ACGAGCCGGAAGCATAAAGTGTAAAG ATCTACAACAGTAGAAATTATTTAAAGTTCTTAGAC
Fnarrayspa2_F	GTCTAAGAACTTTAAATAATTTCTACTGTTGTAGATCTGTACATAACCTTCGGGCATGGCAC
Fnarrayspa2_R	ACTCGTGCCATGCCCGAAGGTTATGTACAGATCTACAACAGTAGAAATTATTTAAAGTTCTTAGACCAGC
Fnarrayspa3'_F	GAGTGTCTAAGAACTTTAAATAATTTCTACTGTTGTAGATCGTATGTTGCATCACCTTCACCCTCT
Fnarrayspa3'_R	CGTTAGAGGGTGAAGGTGATGCAACATACGATCTACAACAGTAGAAATTATTTAAAGTTCTTAGAC
**cF-2/1/3**	**CCCT (5'), GCTG (3'), GAGT (5'), AACG (3')**
Fnarrayspa1_F	CCCTGTCTAAGAACTTTAAATAATTTCTACTGTTGTAGATCGTATGTTGCATCACCTTCACCCTCTGCTG
Fnarrayspa1_R	AGAGGGTGAAGGTGATGCAACATACGATCTACAACAGTAGAAATTATTTAAAGTTCTTAGAC
Fnarrayspa42_F	GTCTAAGAACTTTAAATAATTTCTACTGTTGTAGATCTTTACACTTTATGCTTCCGGCTCGT
Fnarrayspa42_R	ACTCACGAGCCGGAAGCATAAAGTGTAAAGATCTACAACAGTAGAAATTATTTAAAGTTCTTAGACCAGC
Fnarrayspa43_F	GAGTGTCTAAGAACTTTAAATAATTTCTACTGTTGTAGATCTGTACATAACCTTCGGGCATGGCAC
Fnarrayspa43_R	CGTTGTGCCATGCCCGAAGGTTATGTACAGATCTACAACAGTAGAAATTATTTAAAGTTCTTAGAC
**cF-3/1/2**	**CCCT (5'), GCTG (3'), GAGT (5'), AACG (3')**
Fnarrayspa61_F	CCCTGTCTAAGAACTTTAAATAATTTCTACTGTTGTAGATCTGTACATAACCTTCGGGCATGGCACGCTG
Fnarrayspa61_R	GTGCCATGCCCGAAGGTTATGTACAGATCTACAACAGTAGAAATTATTTAAAGTTCTTAGAC
CL375 Fnarrayspa42_F	GTCTAAGAACTTTAAATAATTTCTACTGTTGTAGATCTTTACACTTTATGCTTCCGGCTCGT

CL376 Fnarrayspa42_R	ACTCACGAGCCGGAAGCATAAAGTGTAAAGATCTACAACAGTAGAAATTATTTAAAGTTCTTAGACCAGC
CL316 Fnarrayspa3’_F	GAGTGTCTAAGAACTTTAAATAATTTCTACTGTTGTAGATCGTATGTTGCATCACCTTCACCCTCT
CL317 Fnarrayspa3’_R	CGTTAGAGGGTGAAGGTGATGCAACATACGATCTACAACAGTAGAAATTATTTAAAGTTCTTAGAC
**cF-2/3/1**	**CCCT (5'), GCTG (3'), GAGT (5'), AACG (3')**
CL267 Fnarrayspa1_F	CCCTGTCTAAGAACTTTAAATAATTTCTACTGTTGTAGATCGTATGTTGCATCACCTTCACCCTCTGCTG
CL268 Fnarrayspa1_R	AGAGGGTGAAGGTGATGCAACATACGATCTACAACAGTAGAAATTATTTAAAGTTCTTAGAC
CL269 Fnarrayspa2_F	GTCTAAGAACTTTAAATAATTTCTACTGTTGTAGAT CTGTACATAACCTTCGGGCATGGCAC
CL270 Fnarrayspa2_R	ACTCGTGCCATGCCCGAAGGTTATGTACAGATCTACAACAGTAGAAATTATTTAAAGTTCTTAGACCAGC
CL271 Fnarrayspa3_F	GAGTGTCTAAGAACTTTAAATAATTTCTACTGTTGTAGATCTTTACACTTTATGCTTCCGGCTCGT
CL272 Fnarrayspa3_R	CGTTACGAGCCGGAAGCATAAAGTGTAAAGATCTACAACAGTAGAAATTATTTAAAGTTCTTAGAC
**cF-3/2/1**	**CCCT (5'), GCTG (3'), GAGT (5'), AACG (3')**
CL381 Fnarrayspa61_F	CCCTGTCTAAGAACTTTAAATAATTTCTACTGTTGTAGATCTGTACATAACCTTCGGGCATGGCACGCTG
CL382 Fnarrayspa61_R	GTGCCATGCCCGAAGGTTATGTACAGATCTACAACAGTAGAAATTATTTAAAGTTCTTAGAC
CL379 Fnarrayspa52_F	GTCTAAGAACTTTAAATAATTTCTACTGTTGTAGATCGTATGTTGCATCACCTTCACCCTCT
CL380 Fnarrayspa52_R	ACTCAGAGGGTGAAGGTGATGCAACATACGATCTACAACAGTAGAAATTATTTAAAGTTCTTAGACCAGC
CL271 Fnarrayspa3_F	GAGTGTCTAAGAACTTTAAATAATTTCTACTGTTGTAGATCTTTACACTTTATGCTTCCGGCTCGT
CL272 Fnarrayspa3_R	CGTTACGAGCCGGAAGCATAAAGTGTAAAGATCTACAACAGTAGAAATTATTTAAAGTTCTTAGAC
**cF-4/5/6**	**CCCT (5'), GCTG (3'), GAGT (5'), AACG (3')**
repFnarPlacZ_F	CCCTGTCTAAGAACTTTAAATAATTTCTACTGTTGTAGATCTTTACACTTTATGCTTCCGGCTCGTGCTG
repFnarPlacZ_R	ACGAGCCGGAAGCATAAAGTGTAAAG ATCTACAACAGTAGAAATTATTTAAAGTTCTTAGAC
repFnar1PlacIQ_F	GTCTAAGAACTTTAAATAATTTCTACTGTTGTAGAT GTCGAGTGCAAAACCTTTCGCGGTAT
repFnar1PlacIQ_R	ACTCATACCGCGAAAGGTTTTGCACTCGACATCTACAACAGTAGAAATTATTTAAAGTTCTTAGACCAGC
repFnarParaB_F	GAGTGTCTAAGAACTTTAAATAATTTCTACTGTTGTAGATTCCATACCCGTTTTTTTGGATGGAGT
repFnarParaB_R	CGTTACTCCATCCAAAAAAACGGGTATGGAATCTACAACAGTAGAAATTATTTAAAGTTCTTAGAC
**cF-4/6/5**	**CCCT (5'), GCTG (3'), GAGT (5'), AACG (3')**
repFnarPlacZ_F	CCCTGTCTAAGAACTTTAAATAATTTCTACTGTTGTAGATCTTTACACTTTATGCTTCCGGCTCGTGCTG
repFnarPlacZ_R	ACGAGCCGGAAGCATAAAGTGTAAAGATCTACAACAGTAGAAATTATTTAAAGTTCTTAGAC
repFnar2ParaB_F	GTCTAAGAACTTTAAATAATTTCTACTGTTGTAGATTCCATACCCGTTTTTTTGGATGGAGT

repFnar2ParaB_R	ACTCACTCCATCCAAAAAAACGGGTATGGAATCTACAACAGTAGAAATTATTTAAAGTTCTTAGACCAGC
repFnar2PlacIQ_F	GAGTGTCTAAGAACTTTAAATAATTTCTACTGTTGTAGATGTCGAGTGCAAAACCTTTCGCGGTAT
repFnar2PlacIQ_R	CGTTATACCGCGAAAGGTTTTGCACTCGACATCTACAACAGTAGAAATTATTTAAAGTTCTTAGAC
**cF-5/4/6**	**CCCT (5'), GCTG (3'), GAGT (5'), AACG (3')**
repFnar3PlacIQ_F	CCCTGTCTAAGAACTTTAAATAATTTCTACTGTTGTAGATGTCGAGTGCAAAACCTTTCGCGGTATGCTG
repFnar3PlacIQ_R	ATACCGCGAAAGGTTTTGCACTCGACATCTACAACAGTAGAAATTATTTAAAGTTCTTAGAC
repFnar3PlacZ_F	GTCTAAGAACTTTAAATAATTTCTACTGTTGTAGATCTTTACACTTTATGCTTCCGGCTCGT
repFnar3PlacZ_R	ACTCACGAGCCGGAAGCATAAAGTGTAAAGATCTACAACAGTAGAAATTATTTAAAGTTCTTAGACCAGC
repFnarParaB_F	GAGT GTCTAAGAACTTTAAATAATTTCTACTGTTGTAGAT TCCATACCCGTTTTTTTGGATGGAGT
repFnarParaB_R	CGTTACTCCATCCAAAAAAACGGGTATGGAATCTACAACAGTAGAAATTATTTAAAGTTCTTAGAC
**cFa-1/2/3**	**CCCT (5'), GCTG (3'), GAGT (5'), AACG (3')**
Yeastarray1a_F	CCCTGTCTAAGAACTTTAAATAATTTCTACTGTTGTAGAT GTGCTGACACATACAGGCATATATATGCTG
Yeastarray1a_R	ATATATATGCCTGTATGTGTCAGCAC ATCTACAACAGTAGAAATTATTTAAAGTTCTTAGAC
Yeastarray1b_F	GTCTAAGAACTTTAAATAATTTCTACTGTTGTAGATGAGTCCTCACTCTGAATTCGATATCA
Yeastarray1b_R	ACTCTGATATCGAATTCAGAGTGAGGACTCATCTACAACAGTAGAAATTATTTAAAGTTCTTAGACCAGC
Yeastarray1c_F	GAGT GTCTAAGAACTTTAAATAATTTCTACTGTTGTAGAT CCAGTCACGACGTTGTAAAACGACGG
Yeastarray1c_R	CGTTCCGTCGTTTTACAACGTCGTGACTGGATCTACAACAGTAGAAATTATTTAAAGTTCTTAGAC
**cFa-2/3/1**	**CCCT (5'), GCTG (3'), GAGT (5'), AACG (3')**
Yeastarray2a_F	CCCTGTCTAAGAACTTTAAATAATTTCTACTGTTGTAGATGAGTCCTCACTCTGAATTCGATATCAGCTG
Yeastarray2a_R	TGATATCGAATTCAGAGTGAGGACTC ATCTACAACAGTAGAAATTATTTAAAGTTCTTAGAC
Yeastarray2b_F	GTCTAAGAACTTTAAATAATTTCTACTGTTGTAGATCCAGTCACGACGTTGTAAAACGACGG
Yeastarray2b_R	ACTCCCGTCGTTTTACAACGTCGTGACTGG ATCTACAACAGTAGAAATTATTTAAAGTTCTTAGACCAGC
Yeastarray2c_F	GAGTGTCTAAGAACTTTAAATAATTTCTACTGTTGTAGATGTGCTGACACATACAGGCATATATAT
Yeastarray2c_R	CGTTATATATATGCCTGTATGTGTCAGCACATCTACAACAGTAGAAATTATTTAAAGTTCTTAGAC
**cFa-3/1/2**	**CCCT (5'), GCTG (3'), GAGT (5'), AACG (3')**
Yeastarray3a_F	CCCTGTCTAAGAACTTTAAATAATTTCTACTGTTGTAGATCCAGTCACGACGTTGTAAAACGACGGGCTG
Yeastarray3a_R	CCGTCGTTTTACAACGTCGTGACTGG ATCTACAACAGTAGAAATTATTTAAAGTTCTTAGAC
Yeastarray3b_F	GTCTAAGAACTTTAAATAATTTCTACTGTTGTAGAT GTGCTGACACATACAGGCATATATAT

Yeastarray3b_R	ACTCATATATATGCCTGTATGTGTCAGCACATCTACAACAGTAGAAATTATTTAAAGTTCTTAGACCAGC
Yeastarray3c_F	GAGTGTCTAAGAACTTTAAATAATTTCTACTGTTGTAGATGAGTCCTCACTCTGAATTCGATATCA
Yeastarray3c_R	CGTTTGATATCGAATTCAGAGTGAGGACTCATCTACAACAGTAGAAATTATTTAAAGTTCTTAGAC
**cS-1/2/3**	**CGCA (5'), CCCT (3'), GAGT (5'), TTGC (3')**
newCas9arraylacZ_F	CGCATTTATGCTTCCGGCTCGTATGTTGTGGTTTTAGAGCTATGCTGTTTTGAATGGTCCCAAAACCCCT
newCas9arraylacZ_R	GTTTTGGGACCATTCAAAACAGCATAGCTCTAAAAC CACAACATACGAGCCGGAAGCATAAA
newCas9arraytarg1_F	CAAAGGTGGTCCGCTGCCGTTCGCTTGTTTTAGAGCTATGCTGTTTTGAATGGTCCCAAAAC
newCas9arraytarg1_R	ACTCGTTTTGGGACCATTCAAAACAGCATAGCTCTAAAACAAGCGAACGGCAGCGGACCACCTTTGAGGG
newCas9arraytarg2_F	GAGT ATGGCTAAAAAACCGGTTCAGCTGCC GTTTTAGAGCTATGCTGTTTTGAATGGTCCCAAAAC
newCas9arraytarg2_R	GCAAGTTTTGGGACCATTCAAAACAGCATAGCTCTAAAACGGCAGCTGAACCGGTTTTTTAGCCAT
**cL-3/2/1**	**CCCT (5'), GCTG (3'), GAGT (5'), AACG (3')**
C2C2array43a_F	CCCTGATATAGACCACCCCAATATCGAAGGGGACTAAAACAGGCGATTAAGTTGGGTAACGCCAGGGGCTG
C2C2array43a_R	CCCTGGCGTTACCCAACTTAATCGCCTGTTTTAGTCCCCTTCGATATTGGGGTGGTCTATATC
C2C2array42a_F	GATATAGACCACCCCAATATCGAAGGGGACTAAAACGTAGATGAACTCACCGTCTTGCAGGGA
C2C2array42a_R	ACTCTCCCTGCAAGACGGTGAGTTCATCTACGTTTTAGTCCCCTTCGATATTGGGGTGGTCTATATCCAGC
C2C2array41b_F	GAGTGATATAGACCACCCCAATATCGAAGGGGACTAAAACTAGTCCGGGATGTCAGCCGGGTGTTTA
C2C2array41b_R	CGTTTAAACACCCGGCTGACATCCCGGACTAGTTTTAGTCCCCTTCGATATTGGGGTGGTCTATATC
**c7/8/9/10**	**CCCT (5'), TACA (5'), GCTG (3'), GAGT (5'), AACG (3')**
nnarraySBtoA_F	CCCTTTTATGCTTCCGGCTCGTATGTTGTGGTTTTAGAGCTATGCTGTTTTGAATGGTCCCAAAAC
nnarraySBtoA_R	TGTAGTTTTGGGACCATTCAAAACAGCATAGCTCTAAAACCACAACATACGAGCCGGAAGCATAAA
nnnnarraySAtoB_F	TACACTTTCGCGGTATGGCATGATAGCGCC GTTTTAGAGCTATGCTGTTTTGAATGGTCCCAAAACGCTG
nnnnarraySAtoB_R	GTTTTGGGACCATTCAAAACAGCATAGCTCTAAAACGGCGCTATCATGCCATACCGCGAAAG
arrayPlacZ_F	GTCTAAGAACTTTAAATAATTTCTACTGTTGTAGATCTTTACACTTTATGCTTCCGGCTCGT
arrayPlacZ_R	ACTCACGAGCCGGAAGCATAAAGTGTAAAGATCTACAACAGTAGAAATTATTTAAAGTTCTTAGACCAGC
arrayPLacI_F	GAGTGTCTAAGAACTTTAAATAATTTCTACTGTTGTAGATGTCGAGTGCAAAACCTTTCGCGGTAT
arrayPlacI_R	CGTTATACCGCGAAAGGTTTTGCACTCGACATCTACAACAGTAGAAATTATTTAAAGTTCTTAGAC
**c8/7/9/10**	**CCCT (5'), TACA (5'), GCTG (3'), GAGT (5'), AACG (3')**
Comparr61_F	CCCTCTTTCGCGGTATGGCATGATAGCGCCGTTTTAGAGCTATGCTGTTTTGAATGGTCCCAAAAC

Comparr61_R	TGTAGTTTTGGGACCATTCAAAACAGCATAGCTCTAAAACGGCGCTATCATGCCATACCGCGAAAG
newarraySB_F	TACATTTATGCTTCCGGCTCGTATGTTGTGGTTTTAGAGCTATGCTGTTTTGAATGGTCCCAAAACGCTG
newarraySB_R	GTTTTGGGACCATTCAAAACAGCATAGCTCTAAAACCACAACATACGAGCCGGAAGCATAAA
CL365 arrayPlacZ_F	GTCTAAGAACTTTAAATAATTTCTACTGTTGTAGATCTTTACACTTTATGCTTCCGGCTCGT
CL366 arrayPlacZ_R	ACTCACGAGCCGGAAGCATAAAGTGTAAAGATCTACAACAGTAGAAATTATTTAAAGTTCTTAGACCAGC
CL367 arrayPLacI_F	GAGTGTCTAAGAACTTTAAATAATTTCTACTGTTGTAGATGTCGAGTGCAAAACCTTTCGCGGTAT
CL368 arrayPlacI_R	CGTTATACCGCGAAAGGTTTTGCACTCGACATCTACAACAGTAGAAATTATTTAAAGTTCTTAGAC
**sg/b1/b2**	**TAGC (5'), GAGT (5'), TTCT (3'), AACG (3')**
WAS_F	TAGCTGGATGGAGGAATGAGGAGTGTTTTAGAGCTAGAAATAGCAAGTTAAAATAAGGCTAGTCCG
nWAS_R	ACTCCGGACTAGCCTTATTTTAACTTGCTATTTCTAGCTCTAAAACACTCCTCATTCCTCCATCCA
STKarr1D_F	GAGTGTCTAAGAACTTTAAATAATTTCTACTGTTGTAGATCCACTCCTCATCCCTCCATCCCCTCATTCT
STKarr1D_R	TGAGGGGATGGAGGGATGAGGAGTGGATCTACAACAGTAGAAATTATTTAAAGTTCTTAGAC
GNRHarr1D_F	GTCTAAGAACTTTAAATAATTTCTACTGTTGTAGATTGTACCCACTCCTCATTCCTCCCTCC
GNRHarr1D_R	CGTTGGAGGGAGGAATGAGGAGTGGGTACAATCTACAACAGTAGAAATTATTTAAAGTTCTTAGACAGAA
**sg/b2/b1**	**TAGC (5'), GAGT (5'), TTCT (3'), AACG (3')**
WAS_F	TAGCTGGATGGAGGAATGAGGAGTGTTTTAGAGCTAGAAATAGCAAGTTAAAATAAGGCTAGTCCG
nWAS_R	ACTCCGGACTAGCCTTATTTTAACTTGCTATTTCTAGCTCTAAAACACTCCTCATTCCTCCATCCA
GNRHarr2D_F	GAGTGTCTAAGAACTTTAAATAATTTCTACTGTTGTAGATTGTACCCACTCCTCATTCCTCCCTCCTTCT
GNRHarr2D_R	GGAGGGAGGAATGAGGAGTGGGTACAATCTACAACAGTAGAAATTATTTAAAGTTCTTAGAC
STKarr2D_F	GTCTAAGAACTTTAAATAATTTCTACTGTTGTAGATCCACTCCTCATCCCTCCATCCCCTCA
STKarr2D_R	CGTTTGAGGGGATGGAGGGATGAGGAGTGGATCTACAACAGTAGAAATTATTTAAAGTTCTTAGACAGAA
**cA-t1/t2/t3**	**CCCT (5'), GCTG (3'), GAGT (5'), AACG (3')**
Asarrayspa1_F	CCCTGTCAAAAGACCTTTTTAATTTCTACTCTTGTAGATCACTTTATGCTTCCGGCTCGTATGTTGCTG
Asarrayspa1_R	AACATACGAGCCGGAAGCATAAAGTGATCTACAAGAGTAGAAATTAAAAAGGTCTTTTGAC
Asarrayspa2_F	GTCAAAAGACCTTTTTAATTTCTACTCTTGTAGATTGTCAGTGGAGAGGGTGAAGGTGATG
Asarrayspa2_R	ACTCCATCACCTTCACCCTCTCCACTGACAATCTACAAGAGTAGAAATTAAAAAGGTCTTTTGACCAGC
Asarrayspa3_F	GAGTGTCAAAAGACCTTTTTAATTTCTACTCTTGTAGAT AAGAGTGCCATGCCCGAAGGTTATGT
Asarrayspa3_R	CGTTACATAACCTTCGGGCATGGCACTCTTATCTACAAGAGTAGAAATTAAAAAGGTCTTTTGAC

**cF-2/3/1m1**	**CCCT (5'), GCTG (3'), GAGT (5'), AACG (3')**
pcF-2/3/1m1-fwd	gagtGTCTAAGAACTTTAAATAATTTCTACTGTTGTAGATACATACACTTTATGCTTCCGGCTCGT
pcF-2/3/1m1-rev	cgttACGAGCCGGAAGCATAAAGTGTATGTATCTACAACAGTAGAAATTATTTAAAGTTCTTAGAC
Fnarrayspa1_F	CCCTGTCTAAGAACTTTAAATAATTTCTACTGTTGTAGATCGTATGTTGCATCACCTTCACCCTCTGCTG
Fnarrayspa1_R	AGAGGGTGAAGGTGATGCAACATACGATCTACAACAGTAGAAATTATTTAAAGTTCTTAGAC
Fnarrayspa2_F	GTCTAAGAACTTTAAATAATTTCTACTGTTGTAGATCTGTACATAACCTTCGGGCATGGCAC
Fnarrayspa2_R	actcGTGCCATGCCCGAAGGTTATGTACAGATCTACAACAGTAGAAATTATTTAAAGTTCTTAGACCAGC
**cF-2/3/1m2**	**CCCT (5'), GCTG (3'), GAGT (5'), AACG (3')**
pcF-2/3/1m2-fwd	gagtGTCTAAGAACTTTAAATAATTTCTACTGTTGTAGATCCTTACACTTTATGCTTCCGGCTCGT
pcF-2/3/1m2-rev	cgttACGAGCCGGAAGCATAAAGTGTAAGGATCTACAACAGTAGAAATTATTTAAAGTTCTTAGAC
Fnarrayspa1_F	CCCTGTCTAAGAACTTTAAATAATTTCTACTGTTGTAGATCGTATGTTGCATCACCTTCACCCTCTGCTG
Fnarrayspa1_R	AGAGGGTGAAGGTGATGCAACATACGATCTACAACAGTAGAAATTATTTAAAGTTCTTAGAC
Fnarrayspa2_F	GTCTAAGAACTTTAAATAATTTCTACTGTTGTAGATCTGTACATAACCTTCGGGCATGGCAC
Fnarrayspa2_R	actcGTGCCATGCCCGAAGGTTATGTACAGATCTACAACAGTAGAAATTATTTAAAGTTCTTAGACCAGC
**cF-2/3/1m5**	**CCCT (5'), GCTG (3'), GAGT (5'), AACG (3')**
pcF-2/3/1m3-fwd	gagtGTCTAAGAACTTTAAATAATTTCTACTGTTGTAGATCTTTACACTTTATGCTTCCGGCCAGT
pcF-2/3/1m3-rev	cgttACTGGCCGGAAGCATAAAGTGTAAAGATCTACAACAGTAGAAATTATTTAAAGTTCTTAGAC
Fnarrayspa1_F	CCCTGTCTAAGAACTTTAAATAATTTCTACTGTTGTAGATCGTATGTTGCATCACCTTCACCCTCTGCTG
Fnarrayspa1_R	AGAGGGTGAAGGTGATGCAACATACGATCTACAACAGTAGAAATTATTTAAAGTTCTTAGAC
Fnarrayspa2_F	GTCTAAGAACTTTAAATAATTTCTACTGTTGTAGATCTGTACATAACCTTCGGGCATGGCAC
Fnarrayspa2_R	actcGTGCCATGCCCGAAGGTTATGTACAGATCTACAACAGTAGAAATTATTTAAAGTTCTTAGACCAGC
**cF-2/3/1m4**	**CCCT (5'), GCTG (3'), GAGT (5'), AACG (3')**
pcF-2/3/1m4-fwd	gagtGTCTAAGAACTTTAAATAATTTCTACTGTTGTAGATCTTTACACTTTAGACTTCCGGCTCGT
pcF-2/3/1m4-rev	cgttACGAGCCGGAAGTCTAAAGTGTAAAGATCTACAACAGTAGAAATTATTTAAAGTTCTTAGAC
Fnarrayspa1_F	CCCTGTCTAAGAACTTTAAATAATTTCTACTGTTGTAGATCGTATGTTGCATCACCTTCACCCTCTGCTG
Fnarrayspa1_R	AGAGGGTGAAGGTGATGCAACATACGATCTACAACAGTAGAAATTATTTAAAGTTCTTAGAC
Fnarrayspa2_F	GTCTAAGAACTTTAAATAATTTCTACTGTTGTAGATCTGTACATAACCTTCGGGCATGGCAC

Fnarrayspa2_R	actcGTGCCATGCCCGAAGGTTATGTACAGATCTACAACAGTAGAAATTATTTAAAGTTCTTAGACCAGC
**cF-2/3/1m3**	**CCCT (5'), GCTG (3'), GAGT (5'), AACG (3')**
pcF-2/3/1m5-fwd	gagtGTCTAAGAACTTTAAATAATTTCTACTGTTGTAGATCTTTAACCTTTATGCTTCCGGCTCGT
pcF-2/3/1m5-rev	cgttACGAGCCGGAAGCATAAAGGTTAAAGATCTACAACAGTAGAAATTATTTAAAGTTCTTAGAC
Fnarrayspa1_F	CCCTGTCTAAGAACTTTAAATAATTTCTACTGTTGTAGATCGTATGTTGCATCACCTTCACCCTCTGCTG
Fnarrayspa1_R	AGAGGGTGAAGGTGATGCAACATACGATCTACAACAGTAGAAATTATTTAAAGTTCTTAGAC
Fnarrayspa2_F	GTCTAAGAACTTTAAATAATTTCTACTGTTGTAGATCTGTACATAACCTTCGGGCATGGCAC
Fnarrayspa2_R	actcGTGCCATGCCCGAAGGTTATGTACAGATCTACAACAGTAGAAATTATTTAAAGTTCTTAGACCAGC
**cF-2/3/1m5'**	**CCCT (5'), GCTG (3'), GAGT (5'), AACG (3')**
pcF-2/3/1m7-fwd	gagtGTCTAAGAACTTTAAATAATTTCTACTGTTGTAGATCTTTACACTTTATGCTTCCGGCCATG
pcF-2/3/1m7-rev	cgttCATGGCCGGAAGCATAAAGTGTAAAGATCTACAACAGTAGAAATTATTTAAAGTTCTTAGAC
Fnarrayspa1_F	CCCTGTCTAAGAACTTTAAATAATTTCTACTGTTGTAGATCGTATGTTGCATCACCTTCACCCTCTGCTG
Fnarrayspa1_R	AGAGGGTGAAGGTGATGCAACATACGATCTACAACAGTAGAAATTATTTAAAGTTCTTAGAC
Fnarrayspa2_F	GTCTAAGAACTTTAAATAATTTCTACTGTTGTAGATCTGTACATAACCTTCGGGCATGGCAC
Fnarrayspa2_R	actcGTGCCATGCCCGAAGGTTATGTACAGATCTACAACAGTAGAAATTATTTAAAGTTCTTAGACCAGC
**cF-2/3/1m4'**	**CCCT (5'), GCTG (3'), GAGT (5'), AACG (3')**
pcF-2/3/1m8-fwd	gagtGTCTAAGAACTTTAAATAATTTCTACTGTTGTAGATCTTTACACTCCAGACCCCCGGCTCGT
pcF-2/3/1m8-rev	cgttACGAGCCGGGGGTCTGGAGTGTAAAGATCTACAACAGTAGAAATTATTTAAAGTTCTTAGAC
Fnarrayspa1_F	CCCTGTCTAAGAACTTTAAATAATTTCTACTGTTGTAGATCGTATGTTGCATCACCTTCACCCTCTGCTG
Fnarrayspa1_R	AGAGGGTGAAGGTGATGCAACATACGATCTACAACAGTAGAAATTATTTAAAGTTCTTAGAC
Fnarrayspa2_F	GTCTAAGAACTTTAAATAATTTCTACTGTTGTAGATCTGTACATAACCTTCGGGCATGGCAC
Fnarrayspa2_R	actcGTGCCATGCCCGAAGGTTATGTACAGATCTACAACAGTAGAAATTATTTAAAGTTCTTAGACCAGC
**array library**	**CCCT (5'), GCTG (3'), GAGT (5'), AACG (3')**
Spacer-a1F	ccctGTCTAAGAACTTTAAATAATTTCTACTGTTGTAGATGAAATCGATGCAGAAGGTGTACGTCGgctg
Spacer-a1R	CGACGTACACCTTCTGCATCGATTTCATCTACAACAGTAGAAATTATTTAAAGTTCTTAGAC
Spacer-a2F	ccctGTCTAAGAACTTTAAATAATTTCTACTGTTGTAGATAGCGAGTCGAGCCGCTTCTCGCTTGGgctg
Spacer-a2R	CCAAGCGAGAAGCGGCTCGACTCGCTATCTACAACAGTAGAAATTATTTAAAGTTCTTAGAC

Spacer-a3F	ccctGTCTAAGAACTTTAAATAATTTCTACTGTTGTAGATTCTACGATGCCATTGGGATATATCAAgctg
Spacer-a3R	TTGATATATCCCAATGGCATCGTAGAATCTACAACAGTAGAAATTATTTAAAGTTCTTAGAC
Spacer-a4F	ccctGTCTAAGAACTTTAAATAATTTCTACTGTTGTAGATGGCTGGCGCGAGCCaCTGATGCTCTTgctg
Spacer-a4R	AAGAGCATCAGTGGCTCGCGCCAGCCATCTACAACAGTAGAAATTATTTAAAGTTCTTAGAC
Spacer-a5F	ccctGTCTAAGAACTTTAAATAATTTCTACTGTTGTAGATACCTCGAGGGGATCCTCTAGATTTAAgctg
Spacer-a5R	TTAAATCTAGAGGATCCCCTCGAGGTATCTACAACAGTAGAAATTATTTAAAGTTCTTAGAC
Spacer-b1F	GTCTAAGAACTTTAAATAATTTCTACTGTTGTAGATTTAACCTGCGTGCCTATGCGGATGCA
Spacer-b1R	actcTGCATCCGCATAGGCACGCAGGTTAAATCTACAACAGTAGAAATTATTTAAAGTTCTTAGACcagc
Spacer-b2F	GTCTAAGAACTTTAAATAATTTCTACTGTTGTAGATGTCTAGAGCCTTTTGTATTAGTAGCC
Spacer-b2R	actcGGCTACTAATACAAAAGGCTCTAGACATCTACAACAGTAGAAATTATTTAAAGTTCTTAGACcagc
Spacer-b3F	GTCTAAGAACTTTAAATAATTTCTACTGTTGTAGATAGATTAAAAGGTAATTCTATCTTGTT
Spacer-b3R	actcAACAAGATAGAATTACCTTTTAATCTATCTACAACAGTAGAAATTATTTAAAGTTCTTAGACcagc
Spacer-b4F	GTCTAAGAACTTTAAATAATTTCTACTGTTGTAGATTACCTAGTAGATACGATTACTGATAA
Spacer-b4R	actcTTATCAGTAATCGTATCTACTAGGTAATCTACAACAGTAGAAATTATTTAAAGTTCTTAGACcagc
Spacer-b5F	GTCTAAGAACTTTAAATAATTTCTACTGTTGTAGATTTGTTTGATTGCTTGCATTGAACCTT
Spacer-b5R	actcAAGGTTCAATGCAAGCAATCAAACAAATCTACAACAGTAGAAATTATTTAAAGTTCTTAGACcagc
Spacer-c1F	gagtGTCTAAGAACTTTAAATAATTTCTACTGTTGTAGATATTAGGGTTGGCTGCGTTTCCTCCGT
Spacer-c1R	cgttACGGAGGAAACGCAGCCAACCCTAATATCTACAACAGTAGAAATTATTTAAAGTTCTTAGAC
Spacer-c2F	gagtGTCTAAGAACTTTAAATAATTTCTACTGTTGTAGATGTCTAAACTGGTCGAAATCGACCAGT
Spacer-c2R	cgttACTGGTCGATTTCGACCAGTTTAGACATCTACAACAGTAGAAATTATTTAAAGTTCTTAGAC
Spacer-c3F	gagtGTCTAAGAACTTTAAATAATTTCTACTGTTGTAGATGCGCATTCCAGCAGCGTCAACCCTGC
Spacer-c3R	cgttGCAGGGTTGACGCTGCTGGAATGCGCATCTACAACAGTAGAAATTATTTAAAGTTCTTAGAC
Spacer-c4F	gagtGTCTAAGAACTTTAAATAATTTCTACTGTTGTAGATTTCTAAGGCGAAGGTACCGCCGATAG
Spacer-c4R	cgttCTATCGGCGGTACCTTCGCCTTAGAAATCTACAACAGTAGAAATTATTTAAAGTTCTTAGAC
Spacer-c5F	gagtGTCTAAGAACTTTAAATAATTTCTACTGTTGTAGATGAAGACGGTGGAGTTGTTACCGTTAC
Spacer-c5R	cgttGTAACGGTAACAACtCCACCGTCTTCATCTACAACAGTAGAAATTATTTAAAGTTCTTAGAC

Oligo name	Sequence
newcas9Placz_F	CGCATTTATGCTTCCGGCTCGTATGTTGTGGTTTTAGAGCTATGCTGTTTTGAATGGTCCCAAAAC
newcas9Placz_R	GCAAGTTTTGGGACCATTCAAAACAGCATAGCTCTAAAACCACAACATACGAGCCGGAAGCATAAA
newcas9targ1a_F	CGCACAAAGGTGGTCCGCTGCCGTTCGCTTGTTTTAGAGCTATGCTGTTTTGAATGGTCCCAAAAC
newcas9targ1a_R	GCAAGTTTTGGGACCATTCAAAACAGCATAGCTCTAAAACAAGCGAACGGCAGCGGACCACCTTTG
newcas9targ2b_F	CGCAATGGCTAAAAAACCGGTTCAGCTGCCGTTTTAGAGCTATGCTGTTTTGAATGGTCCCAAAAC
newcas9targ2b_R	GCAAGTTTTGGGACCATTCAAAACAGCATAGCTCTAAAACGGCAGCTGAACCGGTTTTTTAGCCAT
newCas9NT_F	CGCAGTTTTAGAGCTATGCTGTTTTGAATGGTCCCAAAAC
newCas9NT_R	GCAAGTTTTGGGACCATTCAAAACAGCATAGCTCTAAAAC
Q5mAs_F	GTTTATACGTCTCTAACGGTCAAAAGACCTTTTTAATTTCTACTCTTGTAGATAAGCTTGGCTGT
Q5mAs_R	ACAGCCAAGCTTATCTACAAGAGTAGAAATTAAAAAGGTCTTTTGACCGTTAGAGACGTATAAAC
AsPCA1_F	CCCTGTCAAAAGACCTTTTTAATTTCTACTCTTGTAGATCACTTTATGCTTCCGGCTCGTATGTT
AsPCA1_R	CGTTAACATACGAGCCGGAAGCATAAAGTGATCTACAAGAGTAGAAATTAAAAAGGTCTTTTGAC
AsPCA2_F	CCCTGTCAAAAGACCTTTTTAATTTCTACTCTTGTAGATTGTCAGTGGAGAGGGTGAAGGTGATG
AsPCA2_R	CGTTCATCACCTTCACCCTCTCCACTGACAATCTACAAGAGTAGAAATTAAAAAGGTCTTTTGAC
AsPCA3_F	CCCTGTCAAAAGACCTTTTTAATTTCTACTCTTGTAGATAAGAGTGCCATGCCCGAAGGTTATGT
AsPCA3_R	CGTTACATAACCTTCGGGCATGGCACTCTTATCTACAAGAGTAGAAATTAAAAAGGTCTTTTGAC
AsNT_F	CCCTGTCAAAAGACCTTTTTAATTTCTACTCTTGTAGAT
AsNT_R	CGTTATCTACAAGAGTAGAAATTAAAAAGGTCTTTTGAC
ECAsCpf1_F	ATCGGAGCTCAGGAGGACATCATGACACAGTTTGAAGGCTTCACC
ECAsCpf1_R	ATCGAAGCTTTTAGGCGTAGTCGGGCACGT
pua66GFP1s_F	GGTCTTCAATGCTTTGCGAGATGTCCAGACTGCAGGCATG
pua66GFP1s_R	CATGCCTGCAGTCTGGACATCTCGCAAAGCATTGAAGACC
pua66GFP2s_F	AAAATAGGCGTATCACGAGGCCCAGATCATATGAAACAGC
pua66GFP2s_R	GCTGTTTCATATGATCTGGGCCTCGTGATACGCCTATTTT
PLacIQ_F	TCGAGGCCCTTTCGTCTTCTTTCGTCGAGTGCAAAACCTTTCGCGGTATGGCATGATAGCGCCCGGAAGAGAGTCAATTCAGGG
PLacIQ_R	GATCCCCTGAATTGACTCTCTTCCGGGCGCTATCATGCCATACCGCGAAAGGTTTTGCACTCGACGAAAGAAGACGAAAGGGCC
newSB_F	CCCTTTTATGCTTCCGGCTCGTATGTTGTGGTTTTAGAGCTATGCTGTTTTGAATGGTCCCAAAAC

newSB_R	CGTTGTTTTGGGACCATTCAAAACAGCATAGCTCTAAAACCACAACATACGAGCCGGAAGCATAAA
nnnnSAtoB_F	CCCTCTTTCGCGGTATGGCATGATAGCGCCGTTTTAGAGCTATGCTGTTTTGAATGGTCCCAAAAC
nnnnSAtoB_R	CGTTGTTTTGGGACCATTCAAAACAGCATAGCTCTAAAACGGCGCTATCATGCCATACCGCGAAAG
SC_F	CCCTGTCTAAGAACTTTAAATAATTTCTACTGTTGTAGATGTCGAGTGCAAAACCTTTCGCGGTAT
SC_R	CGTTATACCGCGAAAGGTTTTGCACTCGACATCTACAACAGTAGAAATTATTTAAAGTTCTTAGAC
SD_F	CCCTGTCTAAGAACTTTAAATAATTTCTACTGTTGTAGATCTTTACACTTTATGCTTCCGGCTCGT
SD_R	CGTTACGAGCCGGAAGCATAAAGTGTAAAGATCTACAACAGTAGAAATTATTTAAAGTTCTTAGAC
CC22GGfrag3_F	TGGAGCCGGTGAGCGTGGCTCTCGCGGTATCATTGC
CC22GGfrag3_R	TTCACTTTCGGTCTCGAGGGCACTCTATTAATATTTTCGAGATCATTTATCTTTCACTGCGGAGAAGTTTCG
CC22GGfrag1_F	TCGAAAATATTAATAGAGTGCCCTCGAGACCGAAAGTGAAACGTGATTTC
newCC22GGfrag1_R	ATCTACAACAGTAGAAATTATTTAAAGTTCTTAGACCGTTAGAGACCTATAAACGCAGAAAGGCCC
nnCC22GGfrag2_F	CTTTAAATAATTTCTACTGTTGTAGATGCACCGAGTCGGTGGTGC
newCC22GGfrag2_R	GCCACGCTCACCGGCTC
SeqCC22_F1	AGCGGATAACAATTTCACACAGG
SeqCC22_R	CCCAGTCACGACGTTGTAAAACG
SeqCC22_F2	GGCGAACTACTTACTCTAGCTTC
YeastFn1_F	CCCTGTCTAAGAACTTTAAATAATTTCTACTGTTGTAGATGTGCTGACACATACAGGCATATATAT
YeastFn1_R	CGTTATATATATGCCTGTATGTGTCAGCACATCTACAACAGTAGAAATTATTTAAAGTTCTTAGAC
YeastFn2_F	CCCTGTCTAAGAACTTTAAATAATTTCTACTGTTGTAGATGAGTCCTCACTCTGAATTCGATATCA
YeastFn2_R	CGTTTGATATCGAATTCAGAGTGAGGACTCATCTACAACAGTAGAAATTATTTAAAGTTCTTAGAC
YeastFn4_F	CCCTGTCTAAGAACTTTAAATAATTTCTACTGTTGTAGATCCAGTCACGACGTTGTAAAACGACGG
YeastFn4_R	CGTTCCGTCGTTTTACAACGTCGTGACTGGATCTACAACAGTAGAAATTATTTAAAGTTCTTAGAC
YeastFnNT_F	CCCTGTCTAAGAACTTTAAATAATTTCTACTGTTGTAGAT
YeastFnNT_R	CGTTATCTACAACAGTAGAAATTATTTAAAGTTCTTAGAC
WTGFP1s_F	GTTGTAGATCGTATGTTGCATCACCTTCACCCTCTCCACGTCTAAGAAC
WTGFP1s _R	CTTAGACGTGGAGAGGGTGAAGGTGATGCAACATACGATCTACAACAGT
WTGFP2s_F	GTTGTAGATCTGTACATAACCTTCGGGCATGGCACTCTTGTCTAAGAAC
WTGFP2s _R	CTTAGACAAGAGTGCCATGCCCGAAGGTTATGTACAGATCTACAACAGT

pUA66PJ23119fr1_F	CTAGAGTGAAGGTGAAGGTCGTCCGTACGAAGGTACACGTTAAACACCCGGCTGACATCCCGGACTAT
pUA66PJ23119fr1_R	CTAGATAGTCCGGGATGTCAGCCGGGTGTTTAACGTGTACCTTCGTACGGACGACCTTCACCTTCACT
pUA66PJ23119fr2_F	CTAGACTCCTCCCTGCAAGACGGTGAGTTCATCTACTGGACATCACCTCCCACAACGAAGACTACACT
pUA66PJ23119fr2_R	CTAGAGTGTAGTCTTCGTTGTGGGAGGTGATGTCCAGTAGATGAACTCACCGTCTTGCAGGGAGGAGT
pUA66PJ23119fr3_F	CTAGAAAAACCCTGGCGTTACCCAACTTAATCGCCTCTGGAAAGCGGGCAGTGAGCGCAACGCAATTT
pUA66PJ23119fr3_R	CTAGAAATTGCGTTGCGCTCACTGCCCGCTTTCCAGAGGCGATTAAGTTGGGTAACGCCAGGGTTTTT
LshC2C2_F	ATCGGAGCTCAGGAGGACATCATGGGAAATTTATTTGGACATAAGA
LshC2C2_R	ATCGAAGCTTTTATAACGTATCATTCGTATTTTCT
pC2C2GG _F	GATATAGACCACCCCAATATCGAAGGGGACTAAAACAAGCTTGGCTGTTTTGGCGG
pC2C2GG _R	GTTTTAGTCCCCTTCGATATTGGGGTGGTCTATATCCGTTAGAGACGTATAAACGCAGAAAG
C2C21b_F	CCCTGATATAGACCACCCCAATATCGAAGGGGACTAAAACTAGTCCGGGATGTCAGCCGGGTGTTTA
C2C21b_R	CGTTTAAACACCCGGCTGACATCCCGGACTAGTTTTAGTCCCCTTCGATATTGGGGTGGTCTATATC
C2C22a_F	CCCTGATATAGACCACCCCAATATCGAAGGGGACTAAAACGTAGATGAACTCACCGTCTTGCAGGGA
C2C22a_R	CGTTTCCCTGCAAGACGGTGAGTTCATCTACGTTTTAGTCCCCTTCGATATTGGGGTGGTCTATATC
C2C23a_F	CCCTGATATAGACCACCCCAATATCGAAGGGGACTAAAACAGGCGATTAAGTTGGGTAACGCCAGGG
C2C23a_R	CGTTCCCTGGCGTTACCCAACTTAATCGCCTGTTTTAGTCCCCTTCGATATTGGGGTGGTCTATATC
C2C2NT_F	CCCTGATATAGACCACCCCAATATCGAAGGGGACTAAAAC
C2C2NT_R	CGTTGTTTTAGTCCCCTTCGATATTGGGGTGGTCTATATC
dFnaraB1_F	CCCTGTCTAAGAACTTTAAATAATTTCTACTGTTGTAGATTCCATACCCGTTTTTTTGGATGGAGT
dFnaraB1_R	CGTTACTCCATCCAAAAAAACGGGTATGGAATCTACAACAGTAGAAATTATTTAAAGTTCTTAGAC
offGGbkb_F	TAATGCTAGCCGAGACGGAAAGTGAAACGTGAT
offGGbkb_R	TTTCCGTCTCGGCTAGCATTATACCTAGGACTGAG
WAS_F	TAGCTGGATGGAGGAATGAGGAGTGTTTTAGAGCTAGAAATAGCAAGTTAAAATAAGGCTAGTCCG
WAS_R	CGTTCGGACTAGCCTTATTTTAACTTGCTATTTCTAGCTCTAAAACACTCCTCATTCCTCCATCCA
STK25-D_F	TAGCGTCTAAGAACTTTAAATAATTTCTACTGTTGTAGATCCACTCCTCATCCCTCCATCCCCTCA
STK25-D_R	CGTTTGAGGGGATGGAGGGATGAGGAGTGGATCTACAACAGTAGAAATTATTTAAAGTTCTTAGAC
GNRH2-D_F	TAGCGTCTAAGAACTTTAAATAATTTCTACTGTTGTAGATTGTACCCACTCCTCATTCCTCCCTCC
GNRH2-D_R	CGTTGGAGGGAGGAATGAGGAGTGGGTACAATCTACAACAGTAGAAATTATTTAAAGTTCTTAGAC

WASCR4deGFP_F	ATGAGGAGTTGGATGGGTGCGTAAGTGGGTGAATGGATAGGTCCGCAGAGTGGATGTTTGACA
WASCR4deGFP_R	TCCTCCATCCACTCTTACCCATCCATCCAGAGACACAGGGACGGTCAGGTCTTCTGCTGTC
STK25deGFP_F	ATGAGGAGTGGGAAGCTGTTGACTCATGCACATACCTGTCTCCCGCAGAGTGGATGTTTGACA
STK25deGFP_R	CCCTCCATCCCCTCACACACACAAATCATACAGCAACAGGGCGGTCAGGTCTTCTGCTGTC
GNRH2deGFP_F	ATGAGGAGTGGGTACAGAGTTTCATCTGGGGAAGATGAAAAACCGCAGAGTGGATGTTTGACA
GNRH2deGFP_R	TCCTCCCTCCCCCAGCAACCACTATTCTACTTTCTGTCCCTCGGTCAGGTCTTCTGCTGTC
array0mut_F	GCTCGTAACGAAGCTTGGCTGTTTTGGCG
array0mut_R	AGCCAAGCTTCGTTACGAGCCGGAAGCATA
arraymut_F	AAGCTTGGCTGTTTTGGCGG
array2mut_R	CGTTAGAGGGTGAAGGTGATGCAA
SPCqpr108	TTTTGAATGGTTCCAACAAG
SPCqpr109	ATACTTCTATTCTACTCTGAC
ptF-1m1-fwd	TTCCGGCTCGTATGTTGTGTGGAATTGTGAGCG
ptF-1m1-rev	GCATAAAGTGTATGTGAACGGGTGCCTAATGAG
ptF-1m2-fwd	TTCCGGCTCGTATGTTGTGTGGAATTGTGAGCG
ptF-1m2-rev	GCATAAAGTGTAAGGGAACGGGTGCCTAATGAG
ptF-1m5-fwd	TTCCGGCCAGTATGTTGTGTGGAATTGTGAGCG
ptF-1m5-rev	GCATAAAGTGTAAAGGAACGGGTGCCTAATGAG
ptF-1m4-fwd	TTCCGGCTCGTATGTTGTGTGGAATTGTGAGCG
ptF-1m4-rev	GTCTAAAGTGTAAAGGAACGGGTGCCTAATGAG
ptF-1m3-fwd	TTCCGGCTCGTATGTTGTGTGGAATTGTGAGCG
ptF-1m3-rev	GCATAAAGGTTAAAGGAACGGGTGCCTAATGAG
ptF-1m5'-fwd	TTCCGGCCATGATGTTGTGTGGAATTGTGAGCG
ptF-1m5'-rev	GCATAAAGTGTAAAGGAACGGGTGCCTAATGAG
ptF-1m4'-fwd	CCCCGGCTCGTATGTTGTGTGGAATTGTGAGCG
ptF-1m4'-rev	GTCTGGAGTGTAAAGGAACGGGTGCCTAATGAG
CLFrag1_F	GCGGAAAGAACGGTATCAGCTCACTCAAAG

CLFrag1_R	GTCTTGGTACGGTTCACTAAACGAGCTCGT
CLFrag2_F	TTAGTGAACCGTACCAAGACGTCAACTCG
CLFrag2_R	CATTACCCGGGCCAAAACAGCCAAGCTT
CLFrag3_F	CTGTTTTGGCCCGGGTAATGATCAGCCT
CLFrag3_R	GCTGATACCGTTCTTTCCGCCTCAGAAG
CLFrag2MR_R	CATTACCCGGGCCAAAACAGCCAAGCTTAATAATATACAGACAAATTATTTAAAGTTCTTAGACCGTTACACTTTATGCTTCCGG
CLtarg_F	AACAGCCAAGCTTGAAAACTTACCCTTAAATTTAT
CLtarg_R	TTGGCGGATGAGAGAAGTGACAGAAAATTTGTGCC

**Four-nt junctions that can be used to generate CRISPR arrays with up to 9 spacers:**
CCCT
TGGC
TTCT
CAAT
TATG
AGAA
TACA
GCTG
GAGT
AACG

SUPPLEMENTARY FIGURES

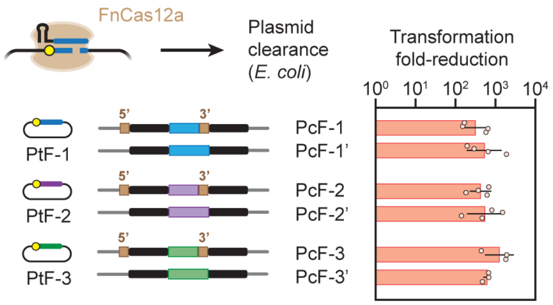

Supplementary Figure 1. Negligible effect of the junction sequences on DNA targeting by FnCas12a. FnCas12a activity was assessed with the plasmid clearance assay described in Figure 3A. As part of the assay, the full-length spacer and spacer containing the junction were tested. Sequences of the spacers and junctions in the assembled arrays are shown in Supplementary Table 4. The spacers are the same as those in Figure 6A. Values represent the average and S.D. of three independent transformation experiments starting from separate colonies. For all three evaluated targets, the fold-reduction in the transformation efficiency were not significantly different in the presence or absence of the junction based on a two-tailed t-test ($p = 0.45$, $n = 4$ for PcF-1/1'; $p = 0.69$, $n = 4$ for PcF-2/2'; $p = 0.29$, $n = 3$ for PcF-3/3'), arguing against any immediate impact of modifying the last four nts of the spacer. Source data are provided as a Source Data file.

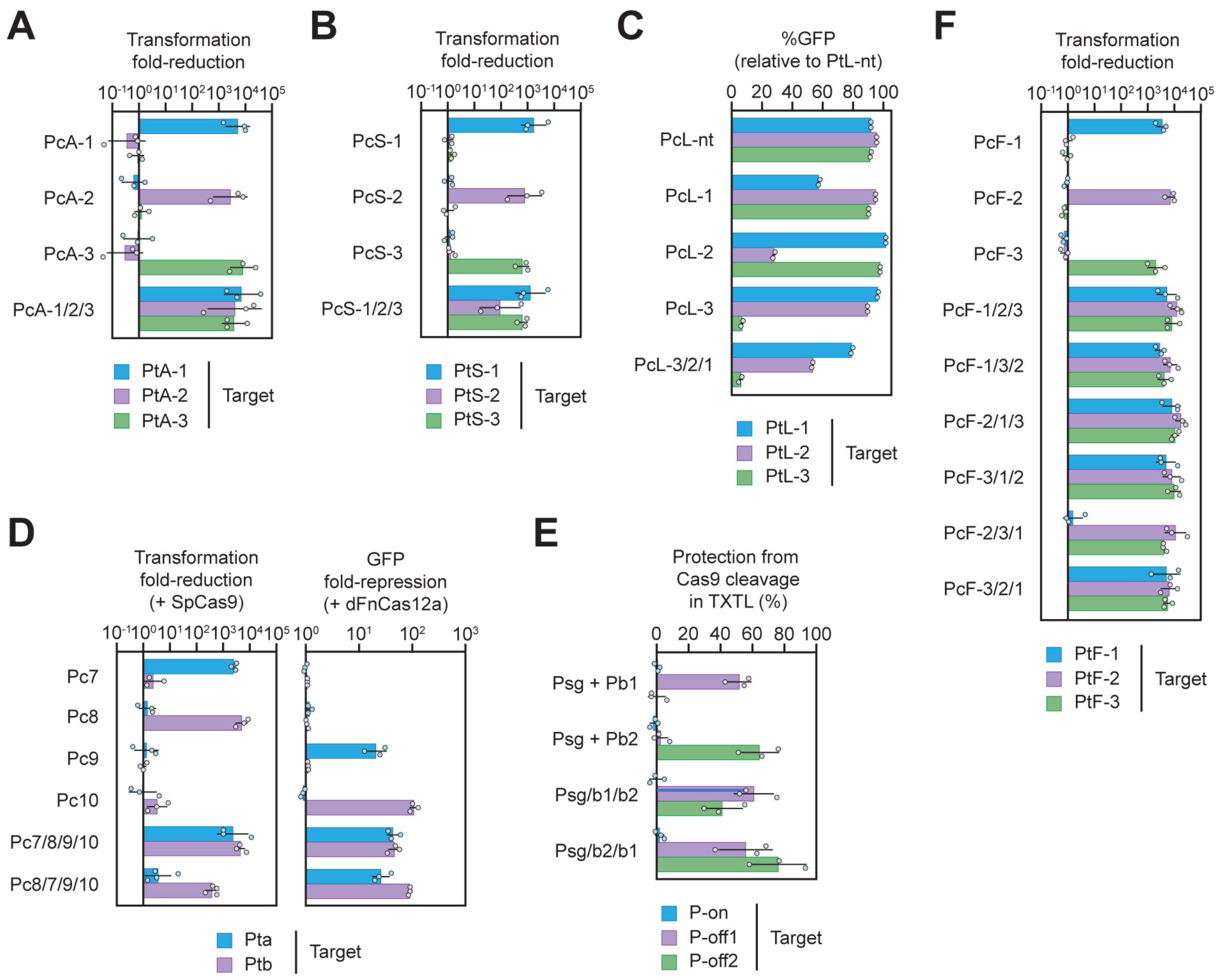

Supplementary Figure 2. Bar graphs for heat maps from the main-text figures. (**A**) From Figure 3A. Values represent the geometric mean and S.D. from three independent experiments starting from separate colonies. (**B**) From Figure 3B. Values represent the geometric mean and S.D. from three independent experiments starting from separate colonies. (**C**) From Figure 3C. Values represent the mean and S.D. from two technical replicates and are representative of three independent experiments. (**D**) From Figure 4C. Values represent the geometric mean and S.D. from at least three independent experiments starting from separate colonies. (**E**) From Figure 4E. Values represent the mean and S.D. from three independent experiments conducted on separate days.

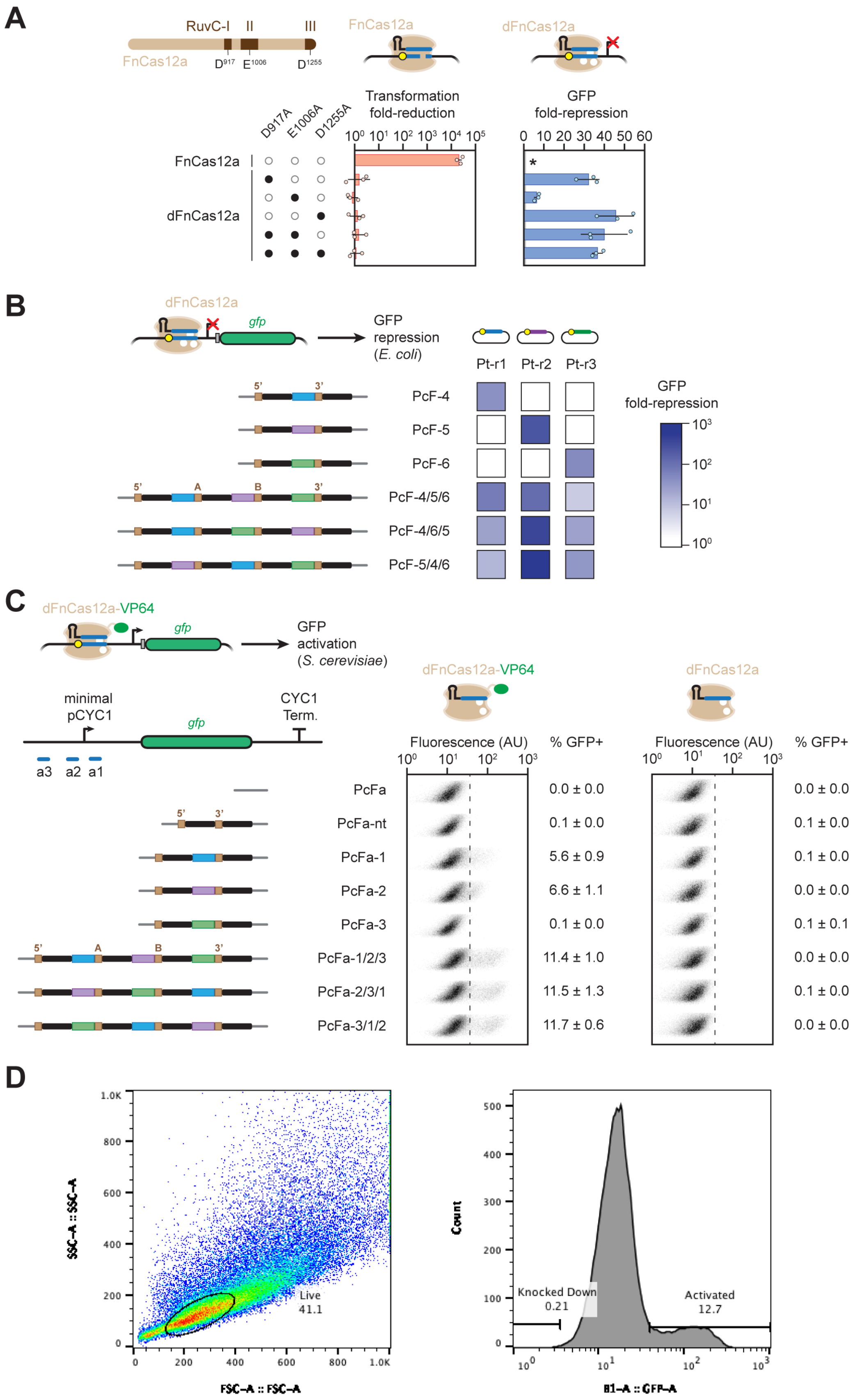
A
RuvC-I
II
III
FnCas12a
D917
E1006
D1255
FnCas12a
dFnCas12a
D917A
E1006A
D1255A
Transformation fold-reduction
GFP fold-repression
FnCas12a
dFnCas12a
B
dFnCas12a
gfp
GFP repression (E. coli)
Pt-r1
Pt-r2
Pt-r3
PcF-4
PcF-5
PcF-6
PcF-4/5/6
PcF-4/6/5
PcF-5/4/6
GFP fold-repression
C
dFnCas12a-VP64
gfp
GFP activation (S. cerevisiae)
minimal pCYC1
CYC1 Term.
a3
a2
a1
dFnCas12a-VP64
dFnCas12a
Fluorescence (AU)
% GFP+
PcFa
PcFa-nt
PcFa-1
PcFa-2
PcFa-3
PcFa-1/2/3
PcFa-2/3/1
PcFa-3/1/2
0.0 ± 0.0
0.1 ± 0.0
5.6 ± 0.9
6.6 ± 1.1
0.1 ± 0.0
11.4 ± 1.0
11.5 ± 1.3
11.7 ± 0.6
0.0 ± 0.0
0.1 ± 0.0
0.1 ± 0.0
0.0 ± 0.0
0.1 ± 0.1
0.0 ± 0.0
0.1 ± 0.0
0.0 ± 0.0
D
SSC-A :: SSC-A
FSC-A :: FSC-A
Live
41.1
Count
B1-A :: GFP-A
Knocked Down
0.21
Activated
12.7

Supplementary Figure 3. Assembled CRISPR-Cas12a arrays allow multiplexed gene regulation in *E. coli* and yeast. (**A**) Impact of RuvC mutations on plasmid clearance and gene repression by FnCas12a in *E. coli*. Transformations were conducted as described in Figure 3A. GFP repression was measured by flow cytometry analysis with cells harboring the dFnCas12a plasmid, a plasmid harboring a targeting or no-spacer array, and the GFP reporter plasmid. Values represent the geometric mean and S.D. of three independent experiments from separate colonies. The WT FnCas12a was not tested in the GFP-repression assay (starred) because of its strong plasmid-clearance activity. (**B**) Multiplexed gene repression with FnCas12a in *E. coli*. See (A) for details, where the triple mutant (D917A, E1006A, D1255A) of FnCas12a was used. Values represent the geometric mean of at least three independent experiments starting from separate colonies. (**C**) Multiplexed gene activation in *S. cerevisiae* with a dFnCas12a-VP64 fusion but not with dFnCas12a. The dFnCas12a double mutant (D917A, E1006A) was used. Values represent the average and S.D. of at least three independent experiments starting from separate colonies. Fluorescence distributions were generated from flow cytometry analysis and plotting fluorescence versus side scatter. (**D**) Gating for the flow cytometry analysis of the yeast cells. Live cells ("live") were gated based on forward scatter versus side scatter, while the percentage of GFP-positive cells (%GFP+) were gated based on the histogram of fluorescence values of live cells ("Activated"). Plots are shown for one replicate with yeast harboring PcFa-2/3/1 and dFnCas12a-VP64. All arrays were assembled using the junctions specified in Supplementary Table 4. Source data are provided as a Source Data file.

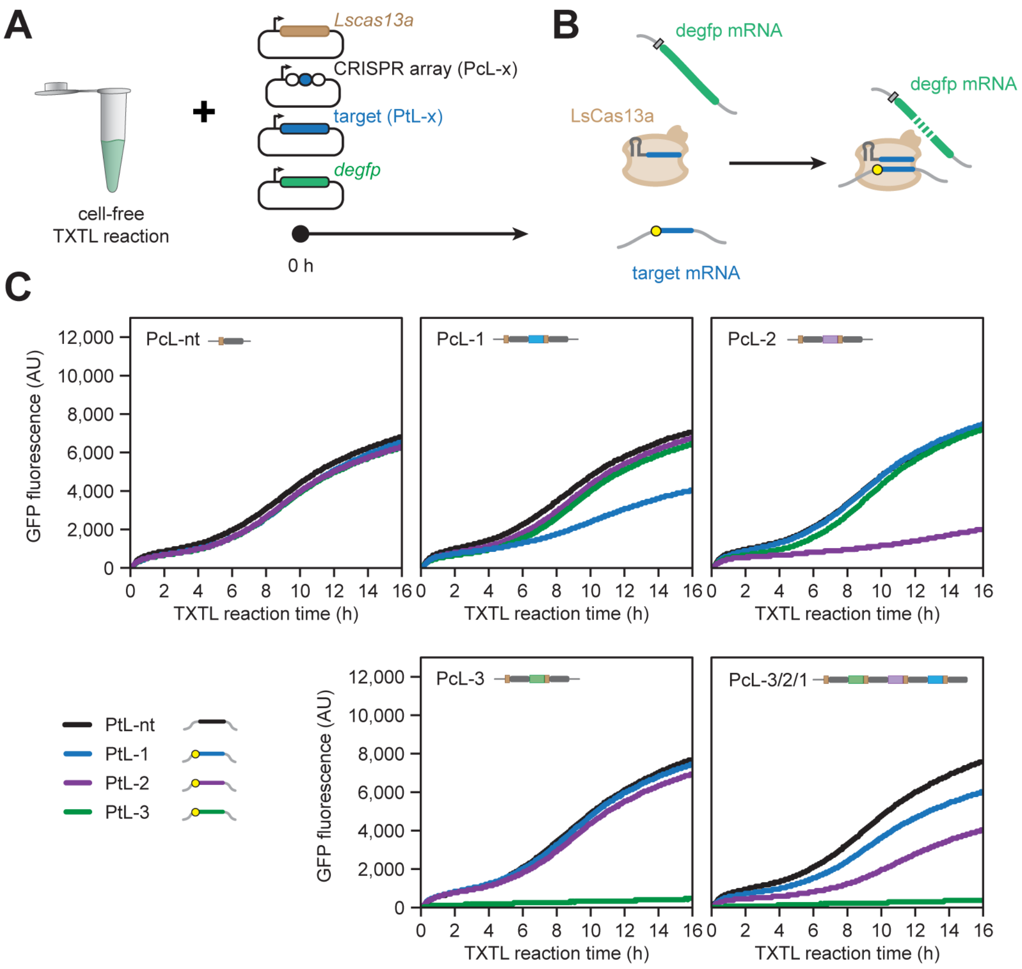

Supplementary Figure 4. Multiplexed RNA sensing in TXTL with LsCas13a. (**A**) All of the DNA components encoding LsCas13a, the CRISPR array, the transcribed target transcript, and the deGFP reporter are added to the TXTL reaction, and GFP fluorescence is tracked over time. (**B**) As part of the reaction, the LsCas13a:crRNA ribonucleoprotein complex recognizes its complementary RNA target flanked by a protospacer-flanking sequence, leading to non-specific degradation of the *degfp* transcript and cessation of deGFP production. (**C**) Representative time courses of GFP fluorescence in TXTL. Each colored line represents a different transcribed target, while each plot represents a different expressed CRISPR array. End-point fluorescence measurements were used to calculate the values shown in Figure 3C and Supplementary Figure 2C.

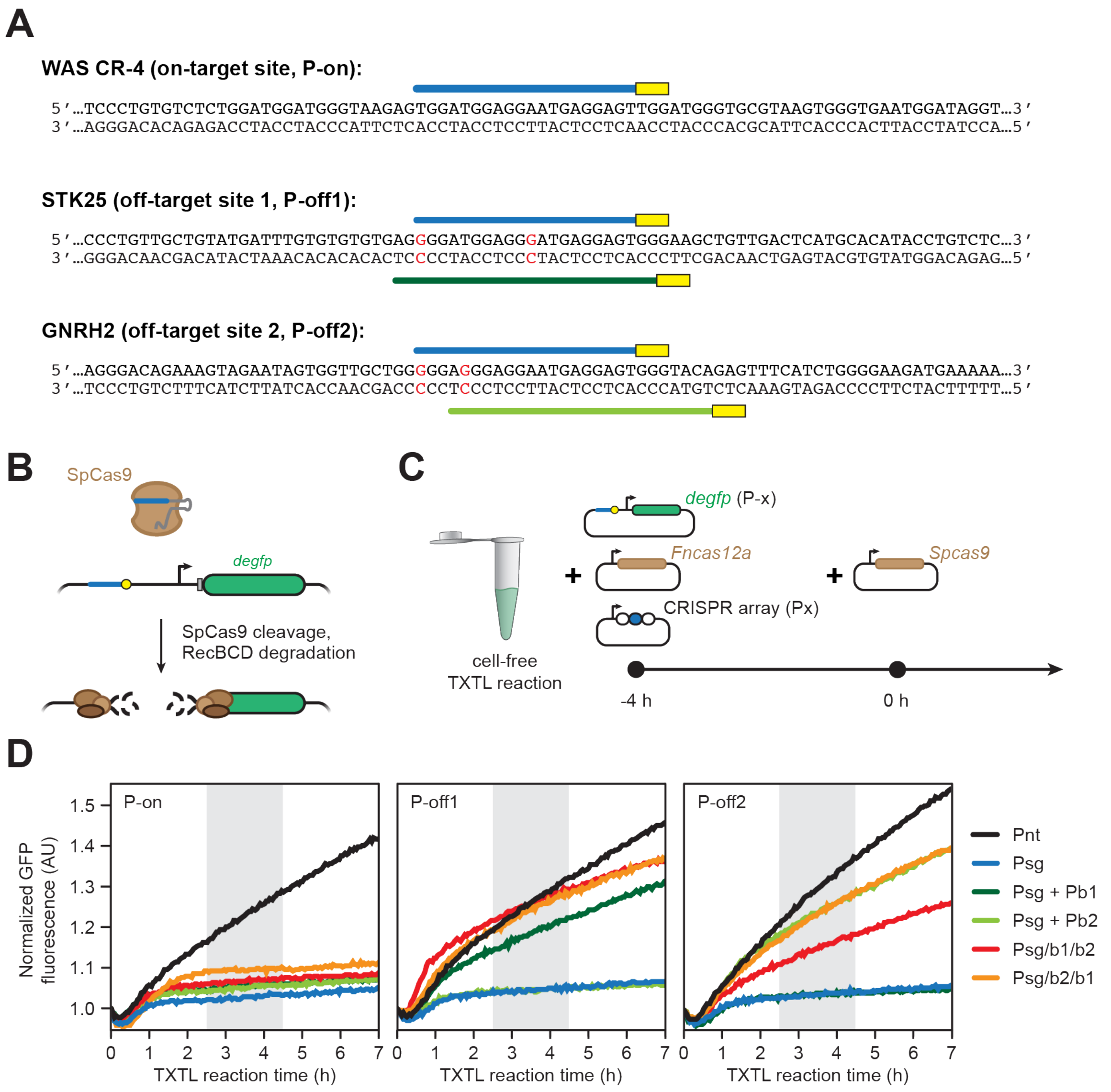

Supplementary Figure 5. Coordinated blocking of known off-target sites in TXTL using composite arrays. (**A**) Selected on-target and off-target sites. An on-target site (WAS CR-4) and the two top off-target sites (STK25, GNRH2) were selected based on prior validation of unintended editing by SpCas9 in human cells[11]. Blue bars correspond to the on-target or off-target sites for the SpCas9 sgRNA, while the green bars represent the targeted blocking sites for the dFnCas12a crRNAs. Yellow boxes correspond to the associated PAMs for SpCas9 (3' NGG) and dFnCas12a (5' YTV). Protospacer colors correspond to the matching spacers in Figure 4D-E. Red letters designate mismatches between the SpCas9 sgRNA and the off-target sequence. (**B**) Mechanism of deGFP repression following target cleavage in TXTL. Plasmid cleavage by SpCas9 allows the linear ends of the DNA to be degraded by RecBCD, resulting in

rapid cessation of deGFP production. Gray box indicates the ribosome-binding site of the *degfp* gene. (**C**) Overview of TXTL experiment. Plasmids encoding the FnCas12a triple mutant (D917A, E1006A, D1255A), a CRISPR array, and the on-target or either off-target sequence upstream of the deGFP reporter construct were combined in the TXTL mix. Four hours later, the plasmid encoding SpCas9 was added, and deGFP fluorescence was tracked over time. The time difference was introduced given the slow kinetics of FnCas12a expression and gRNA binding that we observed previously in TXTL[10,12]. (**D**) Representative time courses of GFP fluorescence in TXTL. Each colored line represents a different CRISPR array, while each plot represents a different target. See Figure 4E for the configuration of each tested CRISPR array. The extent of protection from cleavage by SpCas9 was measured based on GFP production between 2.5 h and 4.5 h into the TXTL reaction followed normalization of the GFP values to the fluorescence at t = 0 h (i.e. addition of the SpCas9 plasmid pCas9). Curves are representative of triplicate experiments conducted independently.

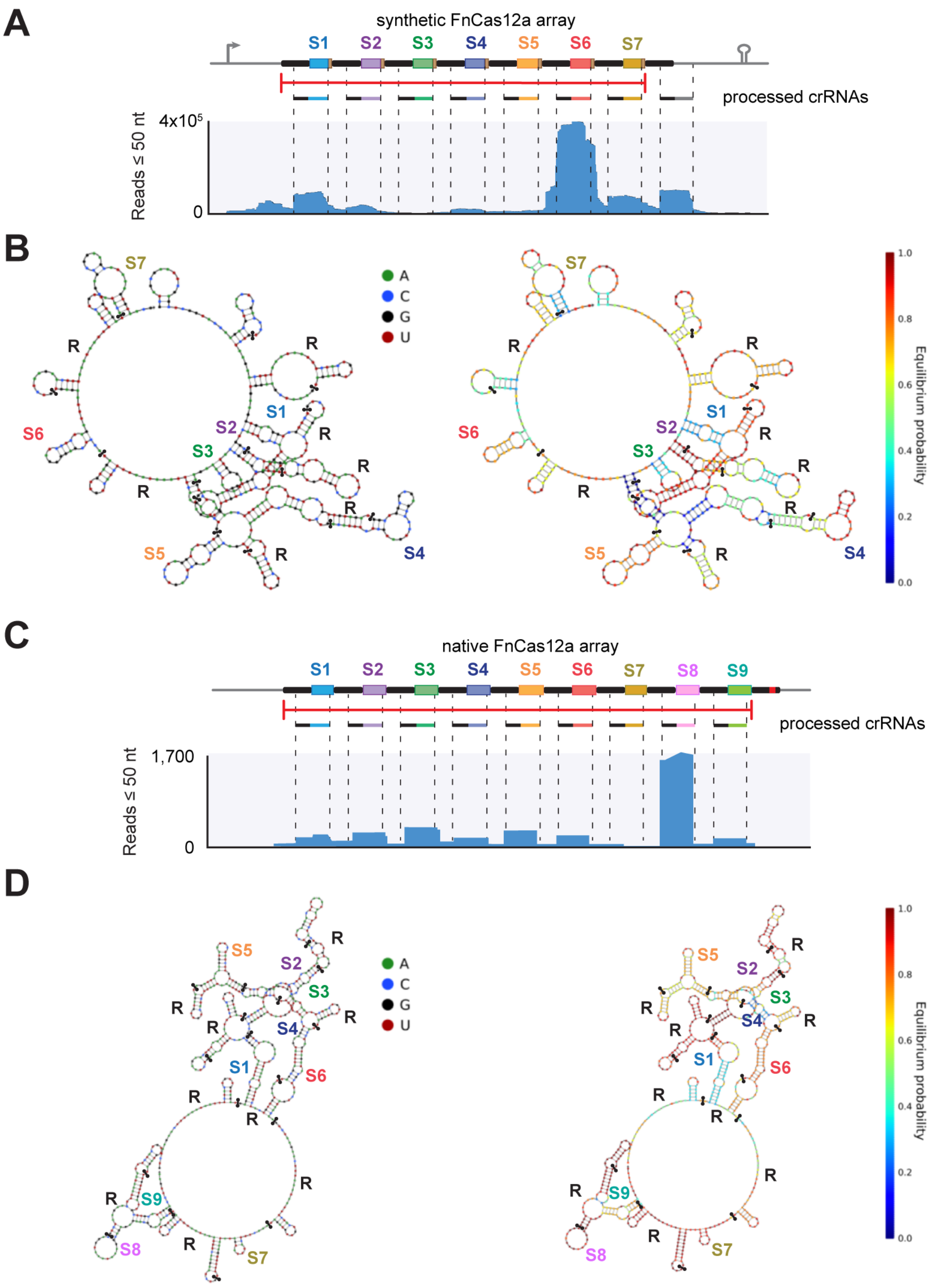

Supplementary Figure 6. Predicted global secondary structure tracks with crRNA abundance. (**A**, **C**) RNA-seq analysis showed variable crRNA abundance from a synthetic and native CRISPR array for FnCas12a. The data for the synthetic seven-spacer array (**A**) is from Figure 5 (top), while the natural nine-spacer array (**C**) is generated from the data presented in Zetsche et al. *Cell* (2015). The red bar below each CRISPR array designates the sequence range used

with the folding predictions. The terminal repeat in the native array is mutated as shown in Figure 7A, explaining the lack of an extraneous CRISPR RNA at the 3' end of the array. (**B**, **D**) Predicted secondary structures with the sequence (left) and base-pairing probabilities (right). Predictions of the minimal-free energy structure and base-pairing probabilities were made using NUPACK (www.nupack.org) for the sequence spanning the regions designed by red bars in (**A**) and (**C**). The regions corresponding to each spacer and repeat are shown. Overall, regions containing stable secondary structures that tended to disrupt the characteristic hairpin in the repeat (S2 - S5 in the synthetic array; S1 - S6, S9 in the native array) were associated with low-abundance processed crRNAs. In contrast, regions lacking a stable secondary structure but formed the characteristic hairpin in the repeat (S1, S6, S7 in the synthetic array; S8 in the native array) — particularly through a repeat flanking the 5' end of a spacer--were associated with the most-abundant crRNAs. The least structured repeat-spacer pairs (S6 in the synthetic array; S8 in the native array) were associated with the most abundant crRNAs. The exception, S7 in the native array, is predicted to harbor a stable 10-bp hairpin between the 3' repeat and the upstream spacer that would be expected to interfere with processing by FnCas12a, where we showed that flanking stable secondary structures can inhibit crRNA processing and resulting nuclease activity[12]. These trends suggest that the global secondary structure of a transcribed array is an important determinant of the final abundance of the processed guide RNAs.

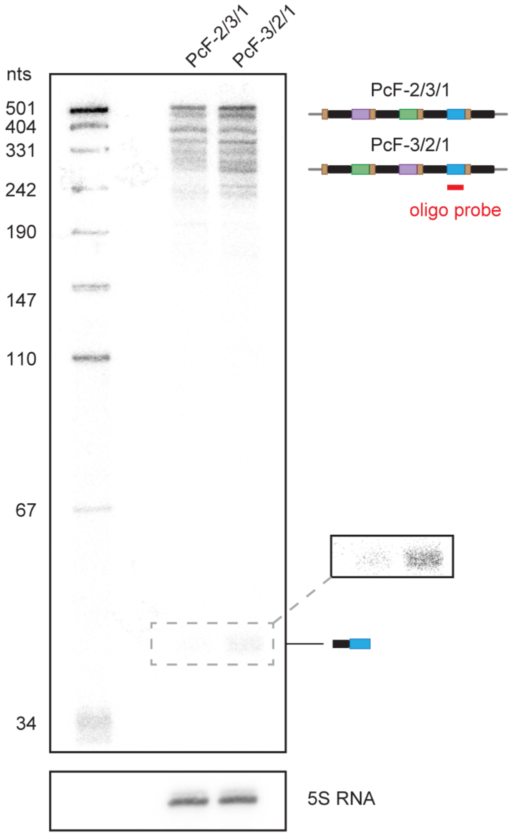

Supplementary Figure 7. Northern blotting analysis of the crRNA associated with spacer S1 from PcF-2/3/1 and PcF-3/2/1 transcribed and processed in TXTL. The gels represent an independent experiment from that in Figure 6C. See Figure 6C for details. Source data are provided as a Source Data file.

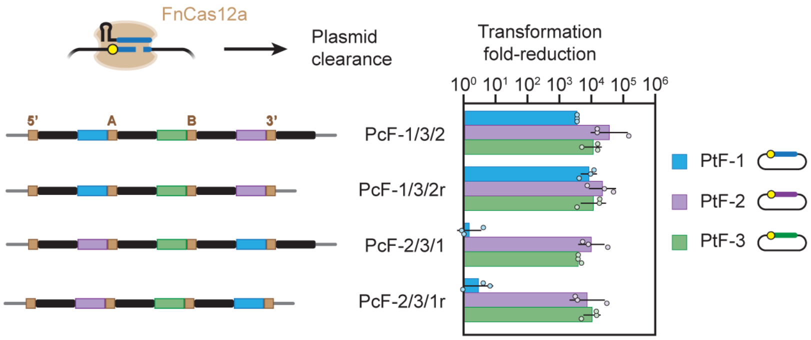

Supplementary Figure 8. Removing the terminal repeat does not rescue plasmid clearance via spacer S1 in PcF-2/3/1. Two, three-spacer CRISPR arrays were selected from Figure 6A based on the robust (PcF-1/3/2) or negligible (PcF-2/3/1) plasmid clearance via spacer S1 (blue). Each array was generated with or without the terminal repeat and subjected to the plasmid clearance assay. See Figure 3A for details on the plasmid clearance assays. Values represent the geometric mean and S.D. from three independent experiments starting from separate colonies. The results show that removal of the terminal repeat did not significantly change plasmid clearance via spacer S1 in PcF-2/3/1 based on a two-tailed t test ($p = 0.48$, $n = 3$). Source data are provided as a Source Data file.

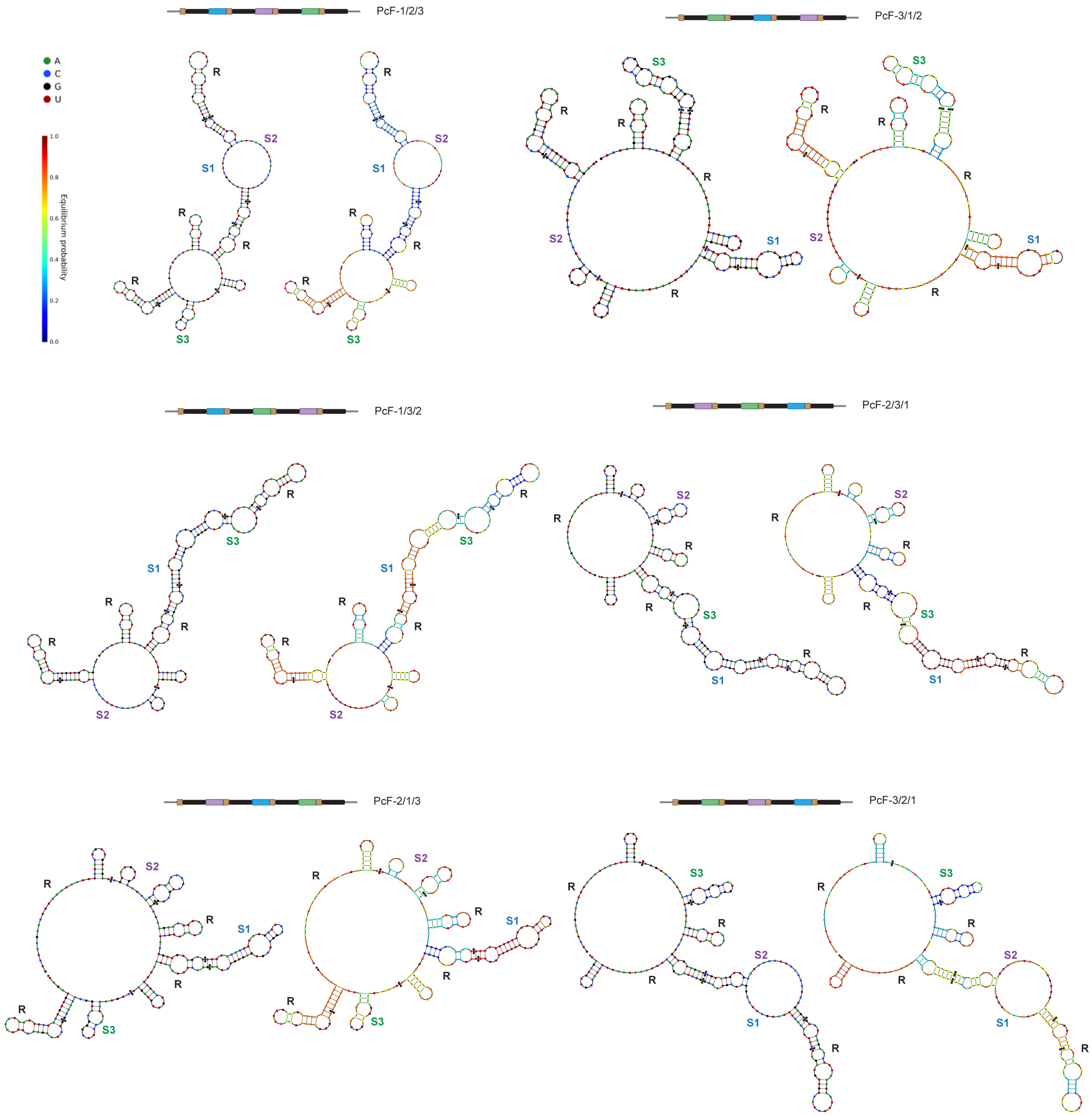
PcF-1/2/3
PcF-3/1/2
PcF-1/3/2
PcF-2/3/1
PcF-2/1/3
PcF-3/2/1
A
C
G
U
1.0
0.8
0.6
0.4
0.2
0.0
Equilibrium probability
S1
S2
S3
R

Supplementary Figure 9. Predicted RNA secondary structures associated with the six permutations of the three-spacer FnCas12a arrays. Arrays are from those in Figure 6A. Predicted secondary structures with the sequence (left) and base-pairing probabilities (right). Predictions of the minimal-free energy structure and base-pairing probabilities were made using NUPACK (www.nupack.org) for each sequence spanning the 5' repeat to the 3' end. All RNAs were predicted to fold into some sort of secondary structure, although spacer S1 in PcF-2/3/1 was the only spacer to be associated with (i) the upstream repeat misfolding into a structure lacking the standard hairpin recognized by FnCas12a and (ii) harboring predicted base pairs with probabilities approaching values of 1.0. The predicted structure associated with PcF-3/2/1 forms a similar imperfect hairpin that includes spacer S1 and its upstream repeat, although the base pairing probabilities are much lower.

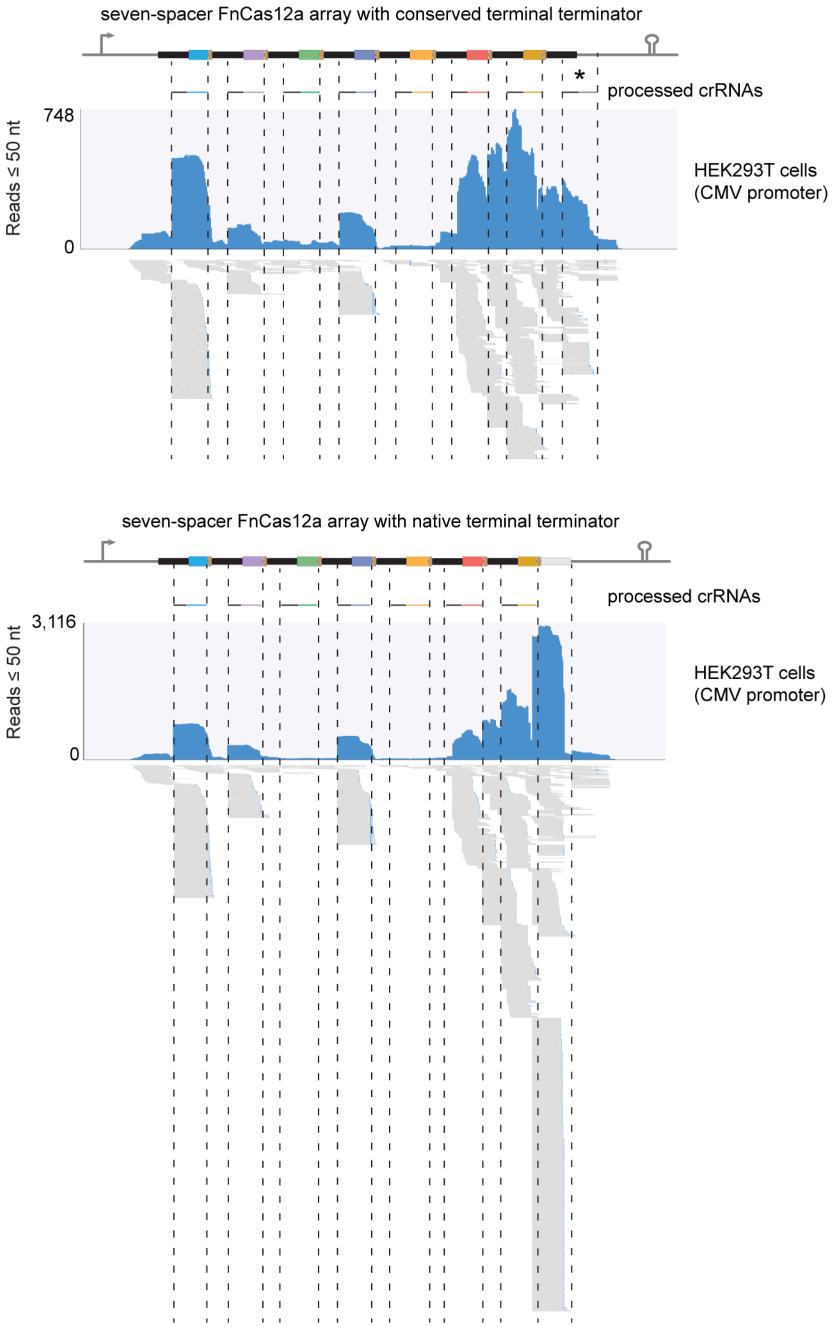

Supplementary Figure 10. RNA-seq analysis of the synthetic seven-spacer array with a consensus repeat (top) or native terminal repeat (bottom) and expressed with FnCas12a in HEK293T cells. The 3' repeat in the seven-spacer array from Figure 5 was replaced with the native terminal repeat from *F. novicida* shown in Figure 7A. RNA-seq analysis was then conducted as described in Figure 5 for HEK293T cells. Each plot shows the compiled (top) or individual (bottom) reads less than 50 nts that mapped to the array expression construct.

SUPPLEMENTARY METHODS

Generation of a CRISPR array using CRATES

This protocol generates a CRISPR array using the CRATES (CRISPR Assembly using Trimmed Ends of Spacers) method. Generation of a three-spacer array targeting three promoters utilized by the Cas12a nuclease from *Francisella novicida* (FnCas12a) is used as an example. Arrays with more sspacers can be generated using the junctions listed in Supplementary Table 4. In our study, arrays with up to seven spacers were generated with a slightly decreased efficiency compare to three-spacer arrays. This method can also be extended to generate array libraries by using the same junctions for the repeat-spacer subunits inserted at the same location in the arrays. The efficiency of the assembly can also be further boosted by slightly modifying the protocol as described below.

Materials

- T4 ligase (New England Biolabs, M0202T)
- T4 ligation buffer (New England Biolabs, B0202S)
- T4 polynucleotide kinase and buffer (New England Biolabs, M0201S)
- Type IIS restriction enzyme BsmBI and buffer (New England Biolabs, R0580S)
- Thermocycler
- TOP10 electrocompetent cells
- SOC liquid medium
- LB agar plates with ampicillin (50 µg/mL)
- Backbone plasmid: pFnCpf1GG (sequence available in Table S2)
- UV flashlight (TaoTronics)

Design oligonucleotides for assembling a three-space CRISPR array

Partial sequences of the promoters targeted by the designed spacers are shown below. The PAM and protospacers are highlighted in yellow and gray, respectively. The protospacer is 30 nts, matching the natural length of spacers in the FnCas12a array.

lacZ promoter	5'…GGCACCCGTTCCTTTACACTTTATGCTTCCGGCTCGTATGTTGTGTGGAATTGT GAGCGGATAACAATT… 3'
lacIQ promoter	5'…TTTCGTCTTCTTTCGTCGAGTGCAAAACCTTTCGCGGTATGGCATGATAGCGCC CGGAAGAGAGTCAATTC… 3'
araB promoter	5'…CTGACGCTTTTTATCGCAACTCTCTACTGTTTCTCCATACCCGTTTTTTTGGATG GAGTGAAACGATGGCGA… 3'

To assemble a three-spacer array with the composition repeat-spacer$_{lacZ}$-repeat-spacer$_{\mathrm{lacIQ}}$ -repeat-spacer$_{araB}$-repeat, oligonucleotides composed of direct repeat and spacer are designed and synthesized. The junctions (CCCT, GCTG, GAGT and AACG) are highlighted in green in the oligonucleotide sequences below. Each junction replaces the 4 nts on the 3' end of the corresponding protospacer—the nts farthest away from the PAM. The first and last junctions are generated when cleaving the GFP-dropout construct with the Type IIS restriction enzyme. The middle junctions alternative between 5' and 3' overhangs to minimize potential mis-assembly of the repeat-spacer subunits.

Repeat-Spacer targeting *lacZ*	Fwd: CCCTGTCTAAGAACTTTAAATAATTTCTACTGTTGTAGATCTTTACACTTTATGCTTC CG GCTCGTGCTG<br><br>Rev: ACGAGCCGGAAGCATAAAGTGTAAAGATCTACAACAGTAGAAATTATTTAAAGTTCT TAGAC
Repeat-Spacer targeting *lacIQ*	Fwd: GTCTAAGAACTTTAAATAATTTCTACTGTTGTAGATGTCGAGTGCAAAACCTTTCGC GGTAT<br><br>Rev: ACTCATACCGCGAAAGGTTTTGCACTCGACATCTACAACAGTAGAAATTATTTAAAG TTCTTAGACCAGC
Repeat-spacer targeting *araB*	Fwd: GAGTGTCTAAGAACTTTAAATAATTTCTACTGTTGTAGATTCCATACCCGTTTTTTTG GATGGAGT<br><br>Rev: CGTTACTCCATCCAAAAAAACGGGTATGGAATCTACAACAGTAGAAATTATTTAAAG TTCTTAGAC

Generation of the array

1. Phosphorylate oligonucleotides using T4 polynucleotide kinase (T4 ligase buffer is used instead of T4 PNK buffer) by adding 41.5 µl water, 5 µl of T4 ligase buffer, 2.5 µl of 10 pM oligonucleotide, and 1 µl of T4 polynucleotide kinase into a PCR tube. Mix by pipetting and incubate at 37°C for 30 minutes in a thermocycler. Then incubate at 65°C for 20 minutes in a thermocycler to heat-inactivate the kinase.

2. To anneal oligonucleotides for making dsDNA bricks for assembly, add 25 µl of the phosphorylated fwd and rev oligonucleotides into a PCR tube and incubate in a thermocycler using the following program: 95°C for 5 min, 94°C for 15 s, decrease by 1°C and hold for 15 seconds for 79 cycles. Note that, for making a library of CRISPR arrays, the holding time for each degree of temperature should be 30 seconds instead of 15 seconds. Place the annealed and phosphorylated oligonucleotides on ice for later use. Note that steps of phosphorylation and annealing can be combined and conducted in one pot to save time.

3. In a PCR tube, add 2 µl of T4 ligation buffer, 1 µl of each annealed repeat-spacer subunit, 50 ng of backbone plasmid (pFnCpf1GG in this case), 1 µl of T4 ligase, 1 µl of BsmBI, and add water to reach a total volume of 20 µl. The ratio of backbone to each repeat-spacer unit should be about 1:20. Mix by pipetting and spin down briefly. Incubate the tube in a thermocycler using the following program: 25 cycles of alternating digestion and ligation (42°C for 2 min, 16°C for 5 min, 25X) followed by a final digestion step (60°C for 10 min) and a heat inactivation step (80°C for 10 min). Note that a total of 35 cycles is used for the one-pot generation of the CRISPR array library.

4. After the incubation step, mix 10 µl of the ligated DNA with 50 µl of water. Then electroporate 1 µl of the diluted ligation reaction into 40 µl of Top10 electrocompetent cells, add 500 µl SOC to the cells, and shake at 250 rpm at 37°C in a culture tube for 1 h. Spread the recovered SOC culture onto an LB plate with appropriate antibiotics (ampicillin for pFnCpf1GG), and incubate overnight at 37 °C.

5. Fluorescent and white colonies can be visually differentiated using a UV flashlight. Screen the white colonies by colony PCR and Sanger sequencing. Sequence of the resulting array

is available from the following link:

https://benchling.com/chunyu/f/ebfIS75s-plasmid-maps-for-multiplexing-manuscript/seq-KnPQEgjT-pcf-456/edit#.

The primers below are for colony PCR and Sanger sequencing when using the pFnCpf1GG as backbone:

Fwd primer:	AGCGCTCATGAGCCCGA
Rev primer:	GGCTGAAAATCTTCTCTCATCCGCC

SUPPLEMENTARY REFERENCES

1. Gomaa, A. A. *et al.* Programmable removal of bacterial strains by use of genome-targeting CRISPR-Cas systems. *MBio* **5,** e00928–13 (2014).
2. Vercoe, R. B. *et al.* Cytotoxic chromosomal targeting by CRISPR/Cas systems can reshape bacterial genomes and expel or remodel pathogenicity islands. *PLoS Genet.* **9,** e1003454 (2013).
3. Cress, B. F. *et al.* CRISPathBrick: Modular combinatorial assembly of Type II-A CRISPR arrays for dCas9-mediated multiplex transcriptional repression in *E. coli*. *ACS Synth. Biol.* **4,** 987–1000 (2015).
4. Zetsche, B. *et al.* Multiplex gene editing by CRISPR-Cpf1 using a single crRNA array. *Nat. Biotechnol.* **35,** 31–34 (2017).
5. Zhang, X. *et al.* Multiplex gene regulation by CRISPR-ddCpf1. *Cell Discov* **3,** 17018 (2017).
6. Abudayyeh, O. O. *et al.* RNA targeting with CRISPR-Cas13. *Nature* **550,** 280–284 (2017).
7. Tak, Y. E. *et al.* Inducible and multiplex gene regulation using CRISPR-Cpf1-based transcription factors. *Nat. Methods* **14,** 1163–1166 (2017).
8. Leenay, R. T. *et al.* Identifying and visualizing functional PAM diversity across CRISPR-Cas systems. *Mol. Cell* **62**, 137–147 (2016).
9. Keung, A. J., Bashor, C. J., Kiriakov, S., Collins, J. J. & Khalil, A. S. Using targeted chromatin regulators to engineer combinatorial and spatial transcriptional regulation. *Cell* **158**, 110–120 (2014).
10. Marshall, R. *et al.* Rapid and scalable characterization of CRISPR technologies using an *E. coli* cell-free transcription-translation system. *Mol. Cell* **69,** 146–157.e3 (2018).

11 Fu, Y. *et al.* High-frequency off-target mutagenesis induced by CRISPR-Cas nucleases in human cells. *Nat. Biotechnol.* **31,** 822–826 (2013).

12. Liao, C., Slotkowski, R. A., Achmedov, T. & Beisel, C. L. The *Francisella novicida* Cas12a

is sensitive to the structure downstream of the terminal repeat in CRISPR arrays. *RNA Biol.* **16**, 404–412 (2019).
