# Supplementary material for: Modular one-pot assembly of CRISPR arrays enables library generation and reveals factors influencing crRNA biogenesis
Source: Nat Commun. 2019 Jul 3;10:2948. doi: 10.1038/s41467-019-10747-3 (PMC6610086; doi:10.1038/s41467-019-10747-3)
Supplement: Supplementary file 3 — Reporting Summary [file 41467_2019_10747_MOESM3_ESM.pdf]

nature research

Corresponding author(s): *Double-blind peer review submissions: write DBPR and your manuscript number here instead of author names.*

Reporting Summary

Nature Research wishes to improve the reproducibility of the work that we publish. This form provides structure for consistency and transparency in reporting. For further information on Nature Research policies, see Authors & Referees and the Editorial Policy Checklist.

Statistical parameters

When statistical analyses are reported, confirm that the following items are present in the relevant location (e.g. figure legend, table legend, main text, or Methods section).

n/a	Confirmed	
☐	☒	The exact sample size (*n*) for each experimental group/condition, given as a discrete number and unit of measurement
☐	☒	An indication of whether measurements were taken from distinct samples or whether the same sample was measured repeatedly
☐	☒	The statistical test(s) used AND whether they are one- or two-sided<br>*Only common tests should be described solely by name; describe more complex techniques in the Methods section.*
☒	☐	A description of all covariates tested
☒	☐	A description of any assumptions or corrections, such as tests of normality and adjustment for multiple comparisons
☒	☐	A full description of the statistics including central tendency (e.g. means) or other basic estimates (e.g. regression coefficient) AND variation (e.g. standard deviation) or associated estimates of uncertainty (e.g. confidence intervals)
☐	☒	For null hypothesis testing, the test statistic (e.g. *F*, *t*, *r*) with confidence intervals, effect sizes, degrees of freedom and *P* value noted<br>*Give P values as exact values whenever suitable.*
☒	☐	For Bayesian analysis, information on the choice of priors and Markov chain Monte Carlo settings
☒	☐	For hierarchical and complex designs, identification of the appropriate level for tests and full reporting of outcomes
☒	☐	Estimates of effect sizes (e.g. Cohen's *d*, Pearson's *r*), indicating how they were calculated
☐	☒	Clearly defined error bars<br>*State explicitly what error bars represent (e.g. SD, SE, CI)*

Our web collection on statistics for biologists may be useful.

Software and code

Policy information about availability of computer code

Data collection	MiSeq machine (Illumina)
Data analysis	Geneious 10.2.3 software package (Biomatters)<br>Salmon on the platform Galaxy Version 0.9.1 (https://usegalaxy.org/)

For manuscripts utilizing custom algorithms or software that are central to the research but not yet described in published literature, software must be made available to editors/reviewers upon request. We strongly encourage code deposition in a community repository (e.g. GitHub). See the Nature Research guidelines for submitting code & software for further information.

Data

Policy information about availability of data

All manuscripts must include a data availability statement. This statement should provide the following information, where applicable:

- Accession codes, unique identifiers, or web links for publicly available datasets
- A list of figures that have associated raw data
- A description of any restrictions on data availability

Accession number for small RNA-seq data are SRP144980 and PRJNA454865. Figure 5, 6B, S6A, and S10 are associated with these data.
Accession number for Next Generation Sequencing of CRISPR array library is PRJNA496034. Figure 2C and 2D are associated with this set of data.

Field-specific reporting

Please select the best fit for your research. If you are not sure, read the appropriate sections before making your selection.

☒ Life sciences ☐ Behavioural & social sciences ☐ Ecological, evolutionary & environmental sciences

For a reference copy of the document with all sections, see nature.com/authors/policies/ReportingSummary-flat.pdf

Life sciences study design

All studies must disclose on these points even when the disclosure is negative.

Sample size	At least three random colonies were picked as biological replication for each assay.
Data exclusions	Experiments were done with at least three replications. All reported data were reproducible.
Replication	Values in the figures represent the average of at least three independent experiments starting from separate colonies or TXTL experiments conducted on separate days.
Randomization	Random colonies of transformants were picked as biological replicates.
Blinding	Blinding is not relevant to the study because no animal or plant were used in this study.

Reporting for specific materials, systems and methods

Materials & experimental systems

n/a	Involved in the study	
☒	☐	Unique biological materials
☒	☐	Antibodies
☐	☒	Eukaryotic cell lines
☒	☐	Palaeontology
☒	☐	Animals and other organisms
☒	☐	Human research participants

Methods

n/a	Involved in the study	
☒	☐	ChIP-seq
☐	☒	Flow cytometry
☒	☐	MRI-based neuroimaging

Eukaryotic cell lines

Policy information about cell lines

Cell line source(s)	Human embryonic kidney 293T (HEK293T) cells
Authentication	Not relevant given the type of experiments we performed.
Mycoplasma contamination	Not relevant given the type of experiments we performed.
Commonly misidentified lines (See ICLAC register)	N/A

Flow Cytometry

Plots

Confirm that:

☒ The axis labels state the marker and fluorochrome used (e.g. CD4-FITC).

☒ The axis scales are clearly visible. Include numbers along axes only for bottom left plot of group (a 'group' is an analysis of identical markers).

☒ All plots are contour plots with outliers or pseudocolor plots.

☒ A numerical value for number of cells or percentage (with statistics) is provided.

Methodology

Sample preparation	Yeast or E. coli cells were grown in liquid medium to specific OD and diluted for reaching appropriate number of events per ml.
Instrument	MACSQuant VYB flow cytometer with 96-well plate sampler was used for measuring the yeast population of GFP expression. Accuri C6 flow cytometer with C6 sampler plate loader (Becton Dickinson) equipped with CFlow plate sampler was used for repression of GFP expression in E. coli
Software	N/A
Cell population abundance	Flow Cytometry was used for measuring the GFP expression of E. coli or yeast, no sorting was done.
Gating strategy	For yeast, the fraction of GFP-positive cells were calculated by setting a threshold on FL1 so <0.1% of the cells lacking a gRNA fall within the the GFP-positive bin. For E. coli, cells stained with DRAQ5 was used to set the specific gate to ensure that no debris appeared within the gate.

☒ Tick this box to confirm that a figure exemplifying the gating strategy is provided in the Supplementary Information.
